# Single‐dose immunisation with a multimerised SARS‐CoV‐2 receptor binding domain (RBD) induces an enhanced and protective response in mice

**DOI:** 10.1002/1873-3468.14171

**Published:** 2021-08-22

**Authors:** Ralf Salzer, Jordan J. Clark, Marina Vaysburd, Veronica T. Chang, Anna Albecka, Leo Kiss, Parul Sharma, Andres Gonzalez Llamazares, Anja Kipar, Julian A. Hiscox, Andrew Owen, A. Radu Aricescu, James P. Stewart, Leo C. James, Jan Löwe

**Affiliations:** ^1^ MRC Laboratory of Molecular Biology Cambridge UK; ^2^ Institute of Infection, Veterinary and Ecological Sciences University of Liverpool UK; ^3^ Laboratory for Animal Model Pathology Institute of Veterinary Pathology Vetsuisse Faculty University of Zurich Switzerland; ^4^ Department of Pharmacology and Therapeutics Centre of Excellence in Long‐acting Therapeutics (CELT) University of Liverpool UK

**Keywords:** coronavirus, COVID‐19, Dps, RBD, SARS‐CoV‐2, subunit vaccine

## Abstract

The COVID‐19 pandemic, caused by the SARS‐CoV‐2 coronavirus, has triggered a worldwide health emergency. Here, we show that ferritin‐like Dps from hyperthermophilic *Sulfolobus islandicus,* covalently coupled with SARS‐CoV‐2 antigens *via* the SpyCatcher system, forms stable multivalent dodecameric vaccine nanoparticles that remain intact even after lyophilisation. Immunisation experiments in mice demonstrated that the SARS‐CoV‐2 receptor binding domain (RBD) coupled to Dps (RBD‐S‐Dps) elicited a higher antibody titre and an enhanced neutralising antibody response compared to monomeric RBD. A single immunisation with RBD‐S‐Dps completely protected hACE2‐expressing mice from serious illness and led to viral clearance from the lungs upon SARS‐CoV‐2 infection. Our data highlight that multimerised SARS‐CoV‐2 subunit vaccines are a highly efficacious modality, particularly when combined with an ultra‐stable scaffold.

## Graphical abstract

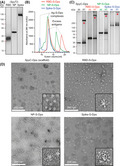

## Abbreviations

**hACE2**, human angiotensin‐converting enzyme 2

**NP**, nucleocapsid protein

**RBD**, receptor binding domain

On 11 March 2020, the World Health Organisation declared the COVID‐19 outbreak, caused by the SARS‐CoV‐2 virus, a pandemic [1]. Since then, COVID‐19 and the efforts to contain it have changed the lives of unprecedented numbers of people. For example, in April 2020 3.9 billion people were affected by lockdown measures aimed to cut or at least reduce the chain of transmission with widespread negative impacts on employment, education, and other health issues. According to the Johns Hopkins University, there have so far been 151 million confirmed COVID‐19 cases globally (May 2021) and virtually every country has been affected. Officially, 3.2 million people have died from SARS‐CoV‐2 infection [2, 3].

**Fig. 1:**
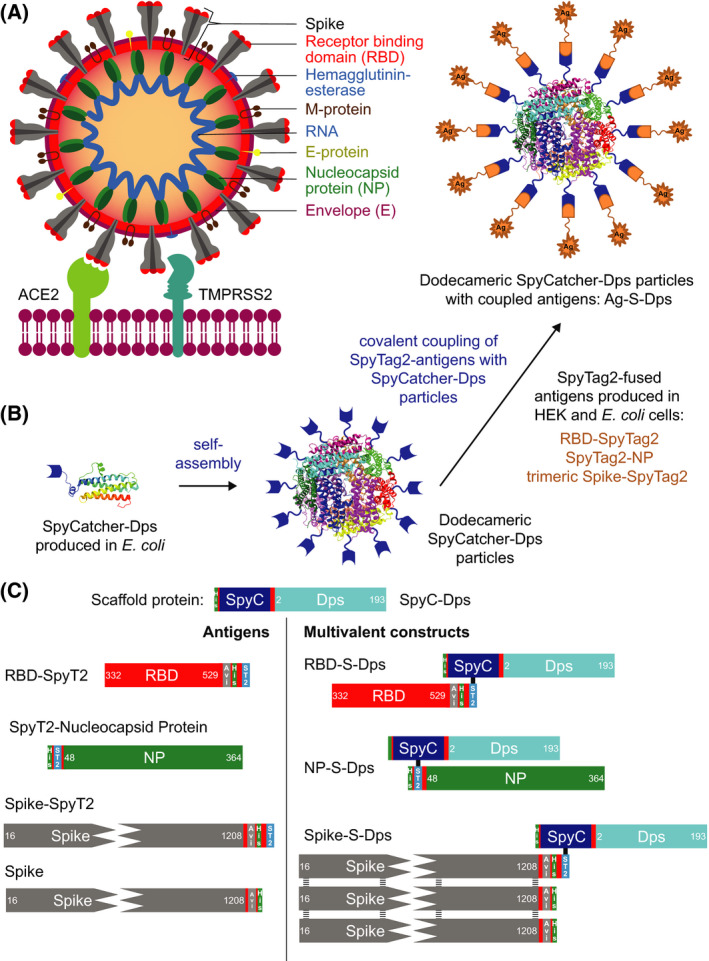
Overview of the multimerisation strategy employed and the antigens and scaffold used. (A) Cartoon representation of SARS‐CoV‐2 binding to a human cell membrane. (B) Schematic diagram of the *Sulfolobus islandicus* Dps and SpyCatcher‐based display and multimerisation strategy employed in this study. (C) Diagram of the proteins used in this work. SpyC is the ΔN1‐SpyCatcher domain, and SpyT2 is the peptidic SpyTag2 that becomes covalently linked to SpyC upon simple mixing. Stabilised, trimeric Spike/Spike‐SpyT2 contained on average only one SpyT2 tag in order to avoid uncontrolled oligomerisation when coupled to Dps.

SARS‐CoV‐2 belongs to the family of Coronaviridae, which contain a positive‐stranded RNA genome [4]. The RNA is enveloped by a membrane that harbours four coat proteins (Fig. 1A). On the inside of the virus, the nucleocapsid protein (NP) is crucial for RNA packaging and viral release from host cells [5]. The Spike protein, which is embedded in the virus' membranous envelope, is essential for the interaction with human angiotensin‐converting enzyme 2 (hACE2) [6]. It is the interaction with hACE2 that is thought to initiate the process that leads to cell entry of viral RNA and infection [7]. The Spike protein is translated as a single polypeptide that is proteolytically processed into its two subunits, S1 and S2. The Spike of SARS‐CoV‐2 is a trimer consisting of three S1‐S2 heterodimers [8]. For membrane fusion between the cell and the virus to occur, two cleavage events within the Spike complex are required [6]. A protease cleavage site located between S1 and S2 is cleaved by the producer cell's proprotein convertase furin during virus assembly [9] (Fig. 1A). The second cleavage site is located in the S2 domain at position R797, and its hydrolysis by the target cell's surface protease TMPRSS2 triggers membrane fusion and cell entry [9].

The SARS‐CoV‐2 receptor binding domain (RBD) is located within the S1 subunit of the Spike. It is the RBD that interacts directly with the host cell *via* the hACE2 receptor [6]. It is therefore not surprising that antibodies directed against the RBD and that overlap with the ACE2 binding region are strongly neutralising, making the RBD a promising subunit vaccine candidate [6, 10]. The RBD is glycosylated and contains four disulphide bridges that contribute to its stability, necessitating its expression in mammalian cells, as is also the case for the Spike.

To end the pandemic, vaccines are by far the most promising approach and vaccine developments, clinical trials, approvals, and mass roll‐outs are in progress. So far, until May 2021, 89 COVID‐19 vaccines have been tested in clinical trials. Of those, 36 are undergoing safety trials, 27 are in the phase of large‐scale testing, 6 vaccines are authorised for limited use, and 8 vaccines are fully approved [2]. All approved vaccines show good‐to‐excellent protection against severe illness, and preliminary data have shown that virus transmission is significantly reduced in vaccinated individuals [11, 12]. Most of the approved vaccines and those in late‐stage trials are mRNA‐based, vector‐based, inactivated viruses or DNA vaccines [11]. Vector‐ and RNA‐based vaccines can often be rapidly developed because they deliver the immunogen coding sequence rather than the immunogen itself. Currently, only one vaccine candidate in late phase trials is a protein‐based subunit vaccine, Novavax [13]. Some subunit vaccines are amenable to processes such as lyophilisation that remove the need for a complex storage and distribution cold‐chain. As such, they provide substantial advantages over nucleic acid‐based vaccines in the quest for complete and global vaccination. A second challenge facing global vaccination is the emergence of viral variants, some of which are more infectious and/or cause more severe illnesses, and reduce the efficacy of existing vaccines [14, 15, 16, 17]. Repeat vaccinations directed against these variants, but that use the same type of vaccine, could be problematic. This is because immunity is generated against the vaccine vector itself, neutralising it before it can deliver its immunogen cargo [18]. It is anticipated that in future, several different types of vaccines will be required to cope with emerging variants of SARS‐CoV‐2.

Previous work has shown that protein‐based subunit vaccines directed against SARS‐CoV‐2 deliver high antibody responses in animal models [19, 20]. Furthermore, subunit antigens have the potential to deliver a cheaper, boostable, and more robust alternative to nucleic acid‐based vaccines [21, 22, 23, 24, 25, 26, 27, 28, 29, 30]. To explore the development of stable and efficient subunit vaccine candidates, here we covalently linked SARS‐CoV‐2 proteins expressed in mammalian and bacterial cells with bacterially expressed Dps from the hyperthermophilic archaeon *Sulfolubus islandicus* [31]. Immunisation using SARS‐CoV‐2 RBD linked to Dps (RBD‐S‐Dps) proved to be highly effective in eliciting an immune response, including after lyophilisation, and to produce neutralising antibodies that inhibit cell entry *in vitro*. Furthermore, transgenic K18‐hACE2 mice infected with SARS‐CoV‐2 were completely protected from serious illness following a single immunisation with RBD‐S‐Dps.

## Materials and methods

### Cloning, expression and purification of the protein components

#### SpyC‐Dps

A hexa‐histidine tag was fused to ΔN1‐SpyCatcher, which was subsequently linked to the Dps from *S*. *islandicus* (ORF SIL_0492, GenBank AGJ61963.1), separated by a GSEGSSGG‐linker (Table S1, SpyC‐Dps). The sequence was codon optimised for the expression in *Escherichia coli,* and the gene was cloned into the pOPINS vector by Gibson assembly. The plasmid encoding for SpyC‐Dps was transformed into C43(DE3) *E. coli*. Cells were grown at 37 °C in 2xYT medium to an OD_600_ of 0.8. Protein production was induced with 1 mm IPTG for 6 h. Cells were harvested at 4500 ***g*** for 25 min at room temperature (RT). Cells were shock‐frozen in liquid nitrogen (LN2) and stored at −80 °C. Cells producing SpyC‐Dps were resuspended in T‐buffer1 (30 mm Tris, 250 mm NaCl, pH 8.0) with one tablet of Complete Protease Inhibitors (Roche, Basel, Switzerland) per 10 g cells wet weight. Cell disruption was carried out using sonication for 7.5 min ‘on’ time, using a 50% duty cycle. Cell debris were removed by centrifugation at 20 000 ***g*** for 30 min at RT. The supernatant was loaded onto a HisTrap FF affinity chromatography column (Cytiva, Freiburg im Breisgau, Germany). Washing was carried out for 17 column volumes (CV) with T‐buffer1 plus 110 mm imidazole. The protein was eluted with T‐buffer1 containing 400 mm imidazole. Purity of fractions was examined by SDS/PAGE, and the purest fraction were pooled and concentrated using a Vivaspin Turbo centrifugal concentrator (100 000 MWCO, Sartorius, Göttingen, Germany). Concentrated sample was loaded onto a size exclusion column (SEC, Sephacryl S‐400, Cytiva), with PBS as the running buffer. Purity was examined by SDS/PAGE, and the sample was frozen in LN2 and stored at −80 °C.

#### SpyT2‐NP

The NP (amino acids 48–364; GenBank: MN908947; NP) was cloned into the vector pOP‐TH and N terminally equipped with a hexa‐histidine tag [32]. A SpyTag2 sequence separated by GS‐rich linkers was inserted between the hexa‐histidine tag and NP (Fig. 1 and Table S1, SpyT2‐NP). The vector encoding for SpyT2‐NP was transformed into *E. coli* C41(DE3) cells. For protein expression, cells were grown at 37 °C in 2xYT medium to an OD_600_ of 0.7. Protein production was induced with 1 mm IPTG for 6 h. Cells were harvested at 4500 ***g*** for 25 min at 4 °C. Cells were frozen in LN2 and stored at −80 °C. SpyT2‐NP‐producing cells were resuspended in T‐buffer2 (50 mm Tris, 1 m NaCl, 10 mm imidazole, 2 mm DTT, pH 8.0) with Complete Protease Inhibitor added (1 tablet per 10 g cells wet weight). Cells were lysed by sonication (3 min total ‘on’ time, duty cycle 50%). Precipitated proteins and cell debris were removed by centrifugation (40 000 ***g***, 1 h, 4 °C). The supernatant was loaded onto a HisTrap FF affinity chromatography column and washed with 20 CV T‐Buffer3 (50 mm Tris, 300 mm NaCl, 1 mm DTT, pH 8.0) containing 20 mm imidazole. Elution was carried out in T‐buffer3 containing 400 mm imidazole. Elution fractions containing NP were loaded onto 20 mL HiTrap Heparin HP column equilibrated in T‐buffer4 (50 Tris, 1 mm DTT, pH 8.0). The column was washed with 3 CV T‐buffer4. Elution was carried out with a linear gradient of 0–2 m NaCl. Elution fractions containing SpyT2‐NP were examined by SDS/PAGE and pooled, and concentrated using a Vivaspin Turbo concentrator with a 10 000 MWCO (Sartorius). Concentrated sample was loaded onto a SEC column (Sephacryl S‐200) (Cytiva) in PBS + 250 mm additional NaCl. Purity was checked by SDS/PAGE, and samples were frozen in LN2 and stored at −80 °C.

#### Spike‐SpyT2 and Spike

To express the ectodomain of the stabilised prefusion Spike protein trimer [33] with only one subunit carrying the SpyTag2 tag, two constructs – one with and one without a SpyTag2 – were made. First, a gene encoding residues 16–1208 of SARS‐CoV‐2 Spike protein (GenBank: MN908947) with proline substitutions at residues 986 and 987, a GSAS substitution at the furin cleavage site (residue 682–685), a C‐terminal T4 fibritin trimerisation motif, a GGSGGS linker, an HRV3C protease cleavage site, a GGS linker and an AviTag, was synthesised and cloned into the lentiviral expression vector pHR‐SFFV [34, 35, 36] downstream of the sequence encoding the chicken RPTPσ secretion signal peptide (cRPTPσSP) [37]. Then, either a GGS linker and a hexa‐histidine tag, or a GGS linker, an octa‐histidine tag, a GGSGGSGGS linker and a SpyTag2 were inserted after the AviTag sequence to form two Spike constructs, with and without a SpyTag2 (Table S1, Spike‐SpyT2 and Spike, respectively). For protein expression and purification, see the next paragraph.

#### RBD‐SpyT2

A gene encoding residue 332–529 of SARS‐CoV‐2 Spike protein (constituting the receptor binding domain, RBD) was synthesised and cloned downstream of cRPTPσ of the pHR‐SFFV vector and a GGSGGS linker, an AviTag, a GGS linker, an octa‐histidine tag, a GGSGGSGGS linker and a SpyTag2 were inserted at the 3′ end of the gene (Table S1, RBD‐SpyT2). The vectors for Spike‐SpyT2, Spike and RBD‐SpyT2 were used for protein production in the mammalian lentiviral expression system [34, 35, 36]. The DNA of the constructs was mixed with the lentiviral envelope and packaging vectors pMD2‐G and psPAX2c (Addgene, Watertown, MA, USA) and polyethylenimine (PEI, Sigma Aldrich, St. Louis, MO, USA) to transiently transfect HEK 293T Lenti‐X cells (Takara Bio, Kusatsu, Japan) to make lentiviral particles. To make Spike trimer protein with only one subunit carrying a SpyTag2, the DNAs of constructs Spike and Spike‐SpyT2 were used at a molar ratio 3 : 1. The virus particles produced were used to infect HEK 293S GnT1^−/−^ cells (for Spike‐SpyT2) or Expi 293 cells (for RBD‐SpyT2). The infected cells were then expanded to obtain 3 L cultures, and conditioned media were harvested and sterile filtered (0.22 μm). The supernatant was concentrated and the buffer exchanged to 25 mm Tris pH 8.0, 300 mm NaCl using an Äkta flux tangential flow system (Cytiva). The conditioned supernatant was then loaded onto a HisTrap column (Cytiva) and washed and eluted with 50 and 250 mm imidazole in the same buffer, respectively. Eluted fractions were checked by SDS/PAGE, pooled and further purified in PBS buffer by SEC on Superdex 200 for RBD and Superose 6 for trimeric Spike protein (both Cytiva). Peak fractions were checked by SDS/PAGE again and frozen in LN2 and stored at −80 °C.

### Coupling and purification of multimerised complexes

For the preparations of Ag‐S‐Dps complexes, comprising RBD‐S‐Dps, NP‐S‐Dps and Spike‐S‐Dps the antigens: RBD‐SpyT2, SpyT2‐NP and Spike/Spike‐SpyT2, and the scaffold protein SpyC‐Dps were diluted in PBS buffer + 250 mm NaCl to 0.2–1 mg·mL^−1^ and mixed. To achieve full occupancy of SpyC‐Dps with the antigens, the molar ratio for SpyC‐Dps to RBD‐SpyT2 was 1 : 1.3, for SpyT2‐NP 1 : 2 and for trimeric Spike/Spike‐SpyT2 1 : 2.5. Reactions were left for ˜ 5 min at RT, and covalent coupling between SpyCather2 and SpyTag2 was checked by SDS/PAGE. Subsequently, samples were concentrated using Vivaspin Turbo concentrators (100 000 MWCO). Antigen‐decorated SpyC‐Dps complexes were separated from the excess antigens by SEC in PBS + 250 mm NaCl on a Superose 6 Increase column (Cytiva). Fractions were checked again for purity by SDS/PAGE, frozen in LN2 and stored at −80 °C.

### Negative‐stain electron microscopy

Proteins were diluted in PBS to concentrations of ˜ 0.012 mg·mL^−1^. Three microlitre of the solution was applied to a glow‐discharged carbon‐coated grid and immediately blotted. For the staining, 10 μL of 2% (w/v) uranyl formate were applied and removed immediately by blotting the grid with filter paper. Images were collected on a FEI Tecnai Spirit 120 kV electron microscope, equipped with a CCD detector.

### *In* *vitro* human plasma stability assay

The *in vitro* stability of RBD‐S‐Dps was studied in clotted human plasma (MD Biomedicals, Taipei City, Taiwan; cat. #2930149). Stocks of the RBD‐S‐Dps samples (751.7 kDa per dodecameric complex) were diluted in PBS to a final concentration of ˜ 0.8 µm and subsequently mixed with prewarmed human plasma in a 1 : 3 (protein : plasma, v/v) ratio. The mixtures were incubated at 37 °C for seven days. Samples were taken after 0, 1, 24, 48, 72, 96, 120 and 168 h, and immediately mixed with denaturing gel‐loading buffer, followed by 30‐min incubation at 99 °C. Inactivated samples were stored at −20 °C before the samples were diluted 1 : 10 with 1× SDS sample buffer and 5 µL per sample were analysed by SDS/PAGE and western blotting. The Ag‐S‐Dps complexes were detected using the HisProbe‐HRP (Thermo Fisher Scientific, Waltham, MA, USA), and human transferrin was used as a loading control and detected using transferrin antibodies from chicken and chicken‐HRP conjugated antibodies (Thermo Fisher Scientific, TFS, cat. #PA1‐9525 and cat. # 31401).

### Lyophilisation of samples

An aliquot of RBD‐S‐Dps of 120 µL (at a protein concentration of 1.4 mg·mL^−1^, in PBS buffer plus additional 250 mm NaCl) was divided into a 40 µL control and a second aliquot of 80 µL. The 80 µL aliquot was lyophilised for 4 h at 30 °C with the aid of a vacuum concentrator (Eppendorf Concentrator 5301) attached to a Savant refrigerated vapor trap (Thermo Fisher Scientific). After lyophilisation to complete dryness, the sample was resuspended in 80 µL Milli‐Q water (Merck KGaA, Darmstadt, Germany). The sample was not centrifuged or processed in any other way after rehydration. EM grids were prepared by staining 1 : 20 and 1 : 100 dilutions (in PBS plus 250 mm NaCl) of lyophilised and resuspended sample with 2% uranyl formate solution on carbon‐coated CF400‐CU‐UL grids (Electron Microscopy Sciences, Hatfield, PA, USA) as described earlier. Imaging was also performed as described earlier. Ten microlitre of lyophilised and rehydrated sample and the untreated control were compared by SDS/PAGE followed by Coomassie staining.

### Mouse immunisation (Fig. 3 and Fig. S1E)

Six‐week‐old C57BL/6J mice (Jackson) were used in immunisation experiments, which were conducted in accordance with the E7 moderate severity limit protocol and the UK Home Office Animals (Scientific Procedures) Act (ASPA, 1986), and approved by the UKRI Animal Welfare and Ethical Review Body. Mice were initially (prime) immunised subcutaneously with 50 µg of the antigens in PBS, mixed with 10 µg CpG ODN 1668 adjuvant (InvivoGen, San Diego, CA, USA). The following antigens were used: RBD‐S‐DPS, RBD‐SpyT2; NP‐S‐DPS, SpyT2‐NP; Spike‐S‐DPS, Spike/Spike‐SpyT2 and SpyC‐Dps. Mice were subcutaneously boosted with 50 µg antigens at day 23 and with 25 µg antigens at day 64. Tail bleeds for ELISA analyses were collected on days 13 and 34.

### Preparation of SARS‐CoV‐2 Spike‐pseudotyped HIV‐1 virions

Replication deficient SARS‐CoV‐2 pseudotyped HIV‐1 virions were prepared as described previously [38]. Briefly, virions were produced in HEK 293T cells by transfection with 1 µg of the plasmid encoding SARS‐CoV‐2 Spike protein (pCAGGS‐SpikeΔc19), 1 µg pCRV GagPol and 1.5 μg GFP‐encoding plasmid (CSGW). Viral supernatants were filtered through a 0.45 μm syringe filter at 48 and 72 h post‐transfection and pelleted for 2 h at 28 000 ***g***. Pelleted virions were drained and then resuspended in DMEM (Gibco, Thermo Fisher Scientific).

### Spike‐pseudotyped neutralisation assays with mouse sera

HEK 293T‐hACE2‐TMPRSS2 cells were described previously [9]. Cells were plated into 96‐well plates at a density of 0.75 × 10^3^ cells per well and allowed to attach overnight. Twenty microlitre pseudovirus‐containing supernatant was mixed with 2 µL dilutions of heat‐inactivated mouse sera and incubated for 40 min at RT. Ten microlitre of this mixture was added to cells. Seventy‐two hour later, cell entry was detected through the expression of GFP by visualisation on an Incucyte S3 live cell imaging system (Sartorius). The per cent of cell entry was quantified as GFP positive areas of cells over the total area covered by cells. Entry inhibition by the sera was calculated as per cent virus infection relative to virus only control.

### ELISA assays

96‐well plates (Nunc) were coated overnight with 5 µg·mL^−1^ of the indicated antigens. Plates were blocked with MPBST: 2% (v/v) milk in PBS, 0.05% Tween‐20. Polyclonal sera from individual mice (challenge experiment) or mouse sera pooled within the same group (mouse immunisation) were diluted as indicated with MPBST and incubated for 45 min on antigen‐coated plates. Plates were washed with MPBST, and bound antibodies were detected with goat anti‐mouse IgG‐HRP (Jackson Immunoresearch, West Grove, PA, USA, #115‐035‐071).

### Cell culture and virus

UK strain of SARS‐CoV‐2 (hCoV‐2/human/Liverpool/REMRQ0001/2020; PANGO lineage B) was used and grown to P4 in Vero E6 cells [39]. The intracellular viral genome sequence and the titre of virus in the stock were determined by direct RNA sequencing (GenBank: MW041156). The virus stock did not contain a deletion of the furin cleavage that has been described previously during passage [40].

### Mouse SARS‐CoV‐2 challenge experiment

Animal work was approved by the local University of Liverpool Animal Welfare and Ethical Review Body and performed under UK Home Office Project Licence PP4715265. Mice carrying the human ACE2 gene under the control of the keratin 18 promoter (K18‐hACE2; formally B6.Cg‐Tg(K18‐ACE2)2Prlmn/J) were purchased from Jackson Laboratories, Bar Harbor, ME, USA. Mice were maintained under SPF barrier conditions in individually ventilated cages. Animals were randomly assigned into multiple cohorts and given 25 µg antigen (RBD‐S‐DPS or RBD‐SpyT2) and 10 µg CpG or PBS *via* subcutaneous injection. On day 28 postimmunisation, mice were anaesthetised lightly with isoflurane and inoculated intranasally with 50 µL containing 10^4^ PFU SARS‐CoV‐2 in PBS. They were culled on day 35 postimmunisation by an overdose of pentabarbitone. Tissues were removed immediately for downstream processing.

### RNA extraction and DNase treatment

The upper lobes of the right lung were dissected and homogenised in 1 mL of TRIzol reagent (TFS) using a Bead Ruptor 24 (Omni International, Kennesaw, GA, USA) at 2 m·s^−1^ for 30 s. The homogenates were clarified by centrifugation at 12 000 ***g*** for 5 min before full RNA extraction was carried out according to manufacturer's instructions. RNA was quantified and quality assessed using a Nanodrop (TFS) before a total of 1 μg was DNase treated using the TURBO DNA‐free kit (TFS) as per manufacturer's instructions.

### qRT‐PCR for viral load

Viral loads were quantified using the GoTaq^®^ Probe 1‐Step RT‐qPCR System (Promega, Madison, WI, USA). For quantification of SARS‐COV‐2, the nCOV_N1 primer/probe mix from the SARS‐CoV‐2 (2019‐nCoV) CDC qPCR Probe Assay (IDT) was utilised whilst the standard curve was generated *via* 10‐fold serial dilution of the 2019‐nCoV_N_Positive Control (IDT) from 10^6^ to 0.1 copies/reaction. The E sgRNA primers and probe have been previously described [41] and were utilised at 400 and 200 nm, respectively. Murine 18S primers and probe sequences were utilised at 400 and 200 nm, respectively. The IAV primers and probe sequences were published as part of the CDC IAV detection kit (20403211; Centre for Disease Control and Prevention, Atlanta, GA, USA). The IAV reverse genetics plasmid encoding the NS segment was diluted 10‐fold from 10^6^ to 0.1 copies/reaction to serve as a standard curve. The thermal cycling conditions for all qRT‐PCRs were as follows: 1 cycle of 45 °C for 15 min and 1 cycle of 95 °C followed by 40 cycles of 95 °C for 15 s and 60 °C for 1 min The 18S standard was generated by the amplification of a fragment of the murine 18S cDNA using the primers F: ACCTGGTTGATCCTGCCAGGTAGC and R: GCATGCCAGAGTCTCGTTCG. Similarly, the E sgRNA standard was generated by PCR using the qPCR primers. cDNA was generated using the SuperScript IV reverse transcriptase kit (TFS) and PCR carried out using Q5 High‐Fidelity 2X Master Mix (New England Biolabs, Ipswich, MA, USA) as per manufacturer's instructions. Both PCR products were purified using the QIAquick PCR Purification Kit (Qiagen, Hilden, Germany) and serially diluted 10‐fold from 10^10^ to 10^4^ copies/reaction to form the standard curve.

### Histology and immunohistology

The left lung lobes were fixed in formal saline for 24 h and routinely paraffin wax embedded. Consecutive sections (3–5 µm) were either stained with haematoxylin and eosin (HE) or used for immunohistology (IH). IH was performed to detect SARS‐CoV‐2 antigen and leukocyte subtypes, that is T cells (CD3+, CD4+, CD8+), B cells (CD45R/B220+) and macrophages (Iba1+), using the horseradish peroxidase (HRP) method and the following primary antibodies: rabbit anti‐SARS‐CoV NP (Rockland Immunochemicals, Limerick, PA, USA, 200‐402‐A50), rabbit anti‐mouse CD3 (clone SP7; Spring Bioscience Corp., Pleasanton, CA, USA), rabbit anti‐mouse CD4 (clone #1; SinoBiological, Beijing, China), rabbit anti‐mouse CD8 (D4W2Z; Cell Signaling Technology, Danvers, MA, USA), rat anti‐mouse CD45R (clone B220; BD Pharmingen Inc, San Diego, CA, USA) and rabbit anti‐human Iba1/AIF1 (FUJIFILM Wako Pure Chemical Corporation, Osaka, Japan; 019‐19741). Briefly, after deparaffination, sections underwent antigen retrieval in citrate buffer (pH 6.0; Agilent Technologies, Santa Clara, CA, USA) (anti‐SARS‐CoV‐2, ‐CD8, ‐CD45R, ‐Iba1) or Tris/EDTA buffer, pH 9.0 (anti‐CD3, anti‐CD4) for 20 min at 98 °C and for 20 min at 37 °C, respectively, followed by incubation with the primary antibody overnight at 4 °C (anti‐SARS‐CoV‐2), 60 min at RT (anti‐CD3, anti‐CD8, anti‐CD45R, anti‐Iba1) or 60 min at 37 °C (anti‐CD3, anti‐CD4). This was followed by blocking of endogenous peroxidase (peroxidase block; Agilent Technologies) for 10 min at RT and incubation with the secondary antibody, EnVision+/HRP, Rabbit and Rat respectively (Agilent Technologies) for 30 min at RT (anti‐SARS‐CoV, anti‐CD8, anti‐CD45R, anti‐Iba1) or the Omni‐Map anti‐Rb HRP (Ventana Medical Systems, Oro Valley, AZ, USA) for 16 min at 37 °C (anti‐CD3, anti‐CD4), followed by EnVision FLEX DAB+ Chromogen in Substrate buffer (Agilent Technologies; anti‐SARS‐CoV‐2, anti‐CD8, anti‐CD45R, anti‐Iba1) for 10 min at RT or the DAB‐Map‐Kit (Ventana; anti‐CD3, ‐CD4), all in an autostainer (Dako Agilent, Santa Clara, CA, USA or Ventana). Sections were subsequently counterstained with haematoxylin.

### ELISpot

ELISpot plates containing PVDF membranes were activated with 15 µL of 35% ethanol for 30 s and washed with distilled water. Plates were then coated overnight at 4 °C with 100 µL of monoclonal antibodies against IFN‐γ 5 µg·mL^−1^ of clone 1‐D1K. ELISpot plates were washed and then blocked with 200 µL R‐10 media for at least 3 h. R‐10 media: RPMI 1640 supplemented with 10% (v/v), FBS, 2 mm l‐glutamine, 100 units penicillin, 0.1 mg·mL^−1^ streptomycin, 10 mm HEPES buffer and 1 mm sodium pyruvate. At the end of incubation, media was discarded and triplicates of 200 000 splenocytes were grown in the presence or absence of Spike peptide pool (PepTivator SARS‐CoV‐2 S peptide pool; Miltenyi Biotec, Bergisch Gladbach, Germany) at 1.5 μg·mL^−1^ final concentration in 100 µL of R‐10 media. After 16‐h incubation at 37 °C, the ELISpot plate was washed followed by incubation with 50 µL biotinylated mouse anti‐mouse IFNγ monoclonal antibody diluted to 0.5 µg·mL^−1^ in 0.5% BSA/PBS for 3 h. Captured IFNγ was detected with 50 µL of anti‐biotin monoclonal antibody and diluted 1 : 750 mL in 0.5% BSA/PBS. After 2 h, the plate was washed, 50 µL of nitro blue tetrazolium/5‐bromo‐4‐chloro‐3‐indolyl‐phosphate was added; purple spots appeared within 10 min. Spot numbers were analysed by an ELISpot reader. Frequencies of Cov‐2 Spike‐specific IFNγ producing cells were calculated by subtracting the number of detected spots in the unstimulated sample from the number of spots detected in the presence of PepTivator SARS‐CoV‐2 protein S peptide pool (average of triplicates), and were given as IFNγ spot forming cells (SFC)/1 × 10^6^ splenocytes.

## Results

### Three multimerised SARS‐CoV‐2 antigen complexes

We aimed to find a stable, convenient, and nonbacterial display scaffold that would allow the display and multimerisation of a range of SARS‐CoV‐2 antigens (Fig. 1A). Multimerisation has been used for many years to increase the immunogenicity of different antigens through multivalency, and this approach has also been shown recently to work well with SARS‐CoV‐2 antigens [20, 21, 25, 28].

For the purpose of stable multimerisation, we identified Dps (ORF SIL_0492) from *S. islandicus*. The source organism is an archaeon, which prefers pH ˜ 3 and, as a hyperthermophile, has adapted to grow optimally at temperatures of around 80 °C. The intrinsic thermostability and environmental robustness of *S. islandicus* Dps make it an outstanding candidate for the development of a multimerisation scaffold. Dps, a member of the ferritin‐like protein family, self‐assembles into hollow, dodecameric spheres with 12 subunits, which are roughly 10 nm across [31]. Most ferritins assemble larger spheres with 24 subunits. Also, in contrast to *bona fide* ferritin scaffolds, both the N and the C termini of Dps are accessible on the outside of the sphere.

**Fig. 2:**
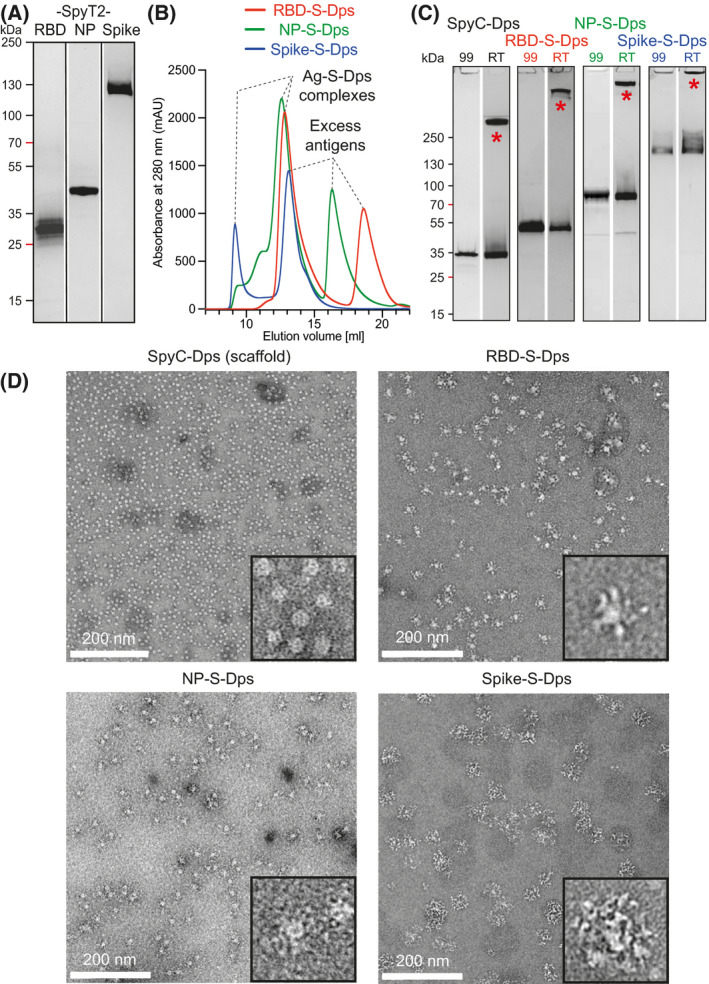
Preparation and quality control of coupled antigen–Dps complexes (Ag‐S‐Dps). (A) SDS/PAGE of the three expressed and purified antigens as introduced in Fig. 1C, Coomassie stained. Glycosylation of Spike leads to a fuzzy appearance of its band. RBD‐SpyT2 and Spike‐SpyT2 were expressed in mammalian cells, and SpyT2‐NP was expressed in bacteria, as was the SpyC‐Dps scaffold. (B) Size exclusion chromatography to separate excess antigens after the SpyCatcher/Spytag2 coupling reactions; Superose 6 Increase in PBS. (C) SDS/PAGE of the coupled and purified Ag‐S‐Dps complexes. ‘RT’, no heating; ‘99’, heated to 99 °C. The SpyC‐Dps scaffold alone, as well as all the three coupled complexes show high‐molecular weight complexes, presumably dodecameric, that disappear only after heating of the samples in SDS loading buffer (Coomassie stained). (D) Negative‐stain electron microscopy analyses of the three multimeric Ag‐S‐Dps complexes, showing that all samples form defined and monodisperse spheres that display the antigens on their surface, leading to particles of different sizes for the three differently sized antigens.

We aimed to test whether Dps could efficiently display Spike, RBD and also NP antigens of SARS‐CoV‐2 (Fig. 1A). Spike and RBD could not be expressed in folded form in *E. coli*, whereas NP as well as Dps expressed and folded well in *E. coli*. Expression of soluble and multimeric antigens genetically fused to Dps in mammalian cells (or *E. coli*) was unsuccessful, and therefore, we decided to employ the SpyCatcher/SpyTag system to attach Dps to different antigens. The SpyCatcher/SpyTag system forms isopeptide bonds between amino acid side chains of the catcher domain and the peptidic tag [42, 43]. ΔN1‐SpyCatcher [44] was fused genetically to the N terminus of Dps, separated by an eight amino acid long GS linker and a hexa‐histidine tag added for purification purposes (SpyC‐Dps, Fig. 1B,C). We chose N‐terminal linkage to Dps, SpyC‐Dps, rather than Dps‐SpyC since the coupling reactions were more efficient, but we did not explore this in any detail. Both the N and C terminus of Dps are on the outside of the sphere and are accessible for covalent coupling. For the antigens, SpyTag2 sequences were fused either at the N or C termini, based on steric considerations (RBD‐SpyT2, SpyT2‐NP, Spike‐SpyT2). Conjugation of stabilised and trimeric Spike‐SpyT2 to the dodecameric SpyC‐Dps could potentially lead to unwanted polymerisation due to the multivalency of both partners. To overcome this problem, and to obtain a biochemically defined sample, we co‐transfected HEK 293T Lenti‐X cells with two different plasmids in a 3 to 1 ratio, one expressing a SpyT2 version and one without SpyT2. This favoured the expression of Spike trimers in which only one of the monomers contains the SpyTag. Stabilised, trimeric, and on average monovalent Spike‐SpyT2 and also RBD‐SpyT2 were purified from conditioned media of HEK 293S GnT1^−/−^ (for Spike‐SpyT2) or Expi 293 (for RBD‐SpyT2) cell cultures. SpyC‐Dps and SpyT2‐NP were purified from the cytosol of *E. coli* cells transformed with the appropriate plasmids. All constructs possess histidine tags and were purified by immobilised metal affinity chromatography and at least one additional size exclusion step (SEC). Sequences of all proteins used can be found in Table S1. Expression yields were excellent in all cases: SpyC‐Dps yielded ˜ 120 mg·L^−1^ culture, RBD‐SpyT2 ˜ 40 mg·L^−1^ culture, stabilised trimeric and monovalent Spike‐SpyT2 ˜ 13 mg·L^−1^ culture and SpyT2‐NP ˜ 60 mg·L^−1^ culture of pure proteins (Fig. 2A).

To achieve efficient coupling of scaffold and antigens, a molar excess of each of the three purified antigens (RBD‐SpyT2, SpyT2‐NP, Spike‐SpyT2) was mixed with SpyC‐Dps to facilitate covalent coupling. Subsequent removal of excess antigens was accomplished by SEC using a Superose 6 column (Fig. 2B). Coupling efficiency was analysed by SDS/PAGE, followed by Coomassie staining (Fig. 2C). When the coupled samples were mixed with denaturating SDS sample buffer without additional heating, we detected high molecular weight complexes that we suggest represent dodecameric assemblies caused by Dps that survive SDS treatment (‘RT’ lanes). Heating the samples to 99 °C led to the disappearance of the higher bands (‘99’ lanes), confirming both the (SDS‐) stability and the purity of the coupled and multimerised protein samples. Note that there were no bands showing uncoupled SpyC‐Dps in any of the three Ag‐S‐Dps samples, meaning that coupling used all 12 available Dps subunits.

Next, we analysed the integrity of the scaffold after the coupling reactions, as well as homogeneity by electron microscopy (Fig. 2D). For the scaffold alone, SpyC‐Dps, we observed the expected small and well‐dispersed ˜ 10 nm Dps spheres. Similar homogeneity and monodispersity were observed for all three coupled Ag‐S‐Dps versions, RBD‐S‐Dps, NP‐S‐Dps and Spike‐S‐Dps. The Ag‐S‐Dps complexes were larger than the scaffold alone as the Dps spheres were densely surrounded by extra densities, indicating the success of the coupling and the structural integrity of Ag‐S‐Dps complexes after the coupling reactions. We note that no aggregation was observed for Spike‐S‐Dps, indicating that the co‐transfection approach produced mostly trimeric Spike proteins with only one SpyTag2 present. Taken together, we showed that the scaffold and the three antigens could be produced easily and at high yields and resulted in biochemically pure and defined Ag‐S‐Dps proteins that display 12 antigens on each Dps scaffold.

To determine whether the coupled Ag‐S‐Dps complexes were stable in blood plasma for immunisations, we mixed the RBD‐S‐Dps complex with human serum (clotted, not heat inactivated, at a 1 : 3 volume ratio). The RBD‐S‐Dps complex was remarkably stable, with 50% remaining intact after ˜ 40 h at 37 °C (Fig. S1A,B). Given the stability of the Dps scaffold both in serum and when exposed to denaturing conditions (SDS/PAGE, ‘RT’ lane) (Fig. 2C), we next investigated whether the coupled RBD‐S‐Dps sample would survive lyophilisation and subsequent re‐solubilisation. A lyophilised, dry sample would facilitate prolonged storage even in the absence of refrigeration. We therefore freeze‐dried RBD‐S‐Dps and after rehydration found no evidence of precipitation or significantly reduced protein concentration by SDS/PAGE (Fig. S1C). There was also no disappearance of the SDS‐stable high molecular weight band, indicating Dps sphere integrity was maintained after re‐hydration. Finally, electron microscopy analysis showed the rehydrated sample to be indistinguishable from the starting material with no evidence of disintegration or aggregation (Fig. S1D).

### Multimerisation by Dps greatly enhances immunogenicity, especially for RBD

**Fig. 3:**
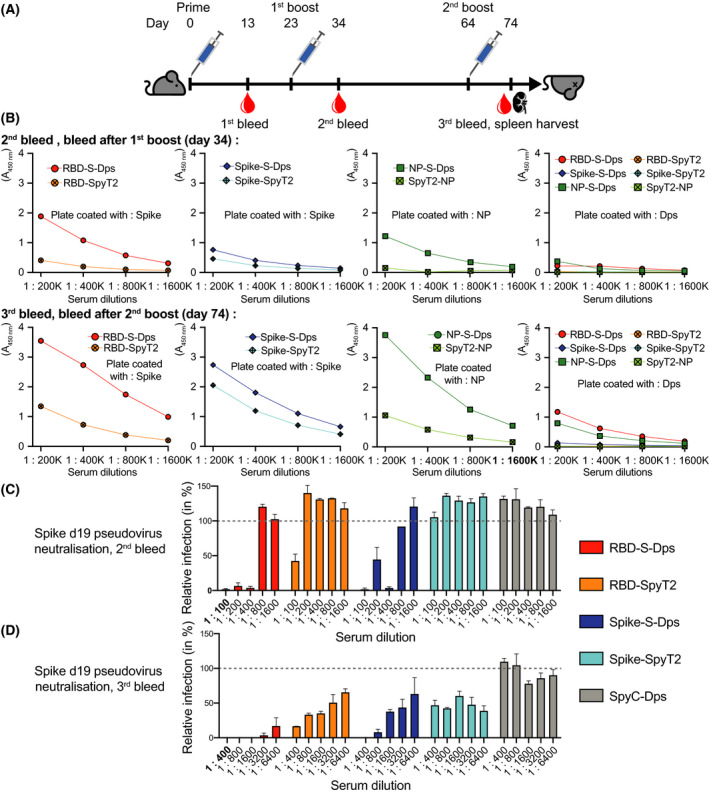
Mouse immunisation – multimeric Ag‐S‐Dps complexes elicit a powerful and neutralising antibody response in mice. (A) Immunisation protocol. (B) Bleeds on day 34 and 74 were tested for binding activity by ELISA against Spike‐SpyT2, NP‐SpyT2 or polymeric scaffold, SpyC‐Dps. In all cases did the multimerised Ag‐S‐Dps complexes produce more antibodies than the nonmultimerised versions. RBD‐S‐Dps and NP‐S‐Dps produced very strong responses. (C) Pseudoviral cell entry neutralisation assay with sera from the 2nd bleed. Sera from immunised mice were tested for neutralisation activity against a Spike‐pseudotyped lentiviral GFP vector (hence NP‐S‐Dps sera will not neutralise). This is a lentiviral vector that incorporates a mutant Spike protein lacking 19 residues from the C terminus (Δ19) into the envelope of the budding particle (see Materials and methods). Infection was measured 72 h after vector addition by quantifying GFP expression in HEK 293T ACE2/TMPRSS2 target cells. The data were normalised to the mean infection level in the absence of virus, which was set to 100%. This is a relative measure and 100% does not indicate that all cells are infected. The multimerised RBD‐S‐Dps and Spike‐S‐Dps showed strong neutralisation, in contrast to their nonmultimerised precursors. (D) Same as (C) but using sera from the 3rd bleed. Neutralisation activity is enhanced in all sera, and the differential between multimerised and nonmultimerised antigens remains. Overall, RBD‐S‐Dps showed the strongest neutralisation activity. Data were measured in triplicates and are given as the mean with error bars representing the standard error of the mean.

Having obtained the three multimerised antigen‐Dps complexes (Ag‐S‐Dps), we tested whether they induce a stronger immune response than their monomeric equivalents. We immunised mice with the following protocol: five male C57BL/6J mice per group were given 50 µg protein subcutaneously on days zero and 23, and 25 µg on day 64 (using CpG 1668 as an adjuvant) (Fig. 3A). Blood samples were taken on days 13 (1st bleed), 34 (2nd bleed), and 74 (3rd bleed). After the 1st boost on day 34, antigen‐specific antibodies were detected in the sera from the mice by ELISA (Fig. 3B). Substantially higher antibody titres were detected with multimerised Dps‐fused RBD and NP. Multimerisation improved Spike titres only modestly, which may be expected given that Spike is already a trimer without Dps. After 74 days, and the second boost, the specific antibody titres were further increased. Spike induced the weakest response and multimerisation had the smallest effect. In contrast, RBD‐S‐Dps and NP‐S‐Dps induced substantial increases in antibody titres compared to the nonmultimerised versions. We also analysed sera for antibodies against the scaffold protein itself (SpyC‐Dps). Sera from mice immunised with coupled Ag‐S‐Dps complexes showed measurable but low antibody titres against SpyC‐Dps. Anti‐SpyC‐Dps responses remained low even after the second boost, suggesting that the scaffold itself is poorly immunogenic and that in the context of the fusions the antibody response is largely directed against the viral antigens displayed on the surface. Furthermore, we tested if lyophilisation affects the ability of RBD‐S‐Dps to induce an anti‐Spike response. Figure S1E shows that single or double lyophilisation do not systematically affect the immunogenicity of RBD‐S‐Dps. Taken together, the data show that multimerised Ag‐S‐Dps complexes produce substantial improvements in antibody titres over the uncoupled antigens. Overall, the strongest response was observed for RBD‐S‐Dps and the strength of the response was not affected by lyophilisation.

Next, we tested the neutralisation activity of antibodies produced by the mice immunised with RBD‐S‐Dps, RBD‐SpyT2, Spike‐S‐Dps and Spike‐SpyT2. The mouse sera within each group were pooled at day 34 (2nd bleed) or 74 (3rd bleed) and analysed using a pseudovirus infection assay (note that NP‐directed sera will not have an effect in this assay because pseudotyped viruses do not contain NP). In this assay, a lentiviral vector expressing GFP is pseudotyped with Spike protein from SARS‐CoV‐2 to obtain virions that display Spike in their envelope and infect cells in an ACE2‐dependent manner. As seen in Fig. 3C, the day 34 sera pool of the multimerised RBD‐S‐Dps group protected against pseudovirus infection up to a dilution of 1 : 400, whereas the monomeric RBD‐SpyT2 only showed a protective effect at a 1 : 100 dilution, and even then, it only reduced infection by ˜ 50%. Sera from mice immunised with multimeric Spike‐S‐Dps also protected against infection, whilst Spike‐SpyT2 sera were unable to neutralise at any of the dilutions tested. The sera taken after 74 days had substantially increased neutralisation activity (Fig. 3D). The sera from RBD‐S‐Dps‐immunised mice gave the strongest protection: even at a 1 : 6400 dilution only ˜ 10% infection could be detected. At this 1 : 6400 dilution, the monomeric RBD‐SpyT2 and Spike‐S‐Dps sera gave very little neutralisation. Whilst pseudoviruses are widely used to test the neutralisation activity of SARS‐CoV‐2 antisera, they are based on a lentiviral rather than coronavirus particle and do not recapitulate live virus replication. We therefore tested whether antibodies raised against multimeric RBD‐S‐Dps were capable of blocking a spreading infection of a primary clinical isolate of SARS‐CoV‐2. Viral replication was measured by RT‐qPCR using probes against *NP* (gRNA) or *E* (sgRNA). RBD‐S‐Dps antisera from five different animals all potently inhibited SARS‐CoV‐2 (Fig. S2A,B). In contrast, the potency of antisera raised against RBD‐SpyT2 varied considerably between mice. We conclude that immunisation with RBD‐S‐Dps not only produces the highest titre antibodies (Fig. 3B), but also the most neutralising (Fig. 3C,D) and with reliable potency against live virus (Fig. S2A,B). We also used ELISpot to test splenocytes from immunised mice culled on day 74 for their T‐cell responses. We observed a statistically significant increase in the proportion of Spike‐specific splenocytes in RBD‐S‐Dps immunised mice versus SpyC‐Dps alone, but not between RBD‐SpyT2 and SpyC‐Dps alone, or between RBD‐S‐Dps and RBD‐SpyT2 (Fig. S2C). However, given the relatively weak response, we reasoned it would not be possible to properly assess whether multimerisation improves T‐cell immunity with this version of the Dps scaffold and so focussed on the antibody response.

### Single‐shot immunisation with multimerised RBD‐S‐Dps protects mice against SARS‐CoV‐2 infection

**Fig. 4:**
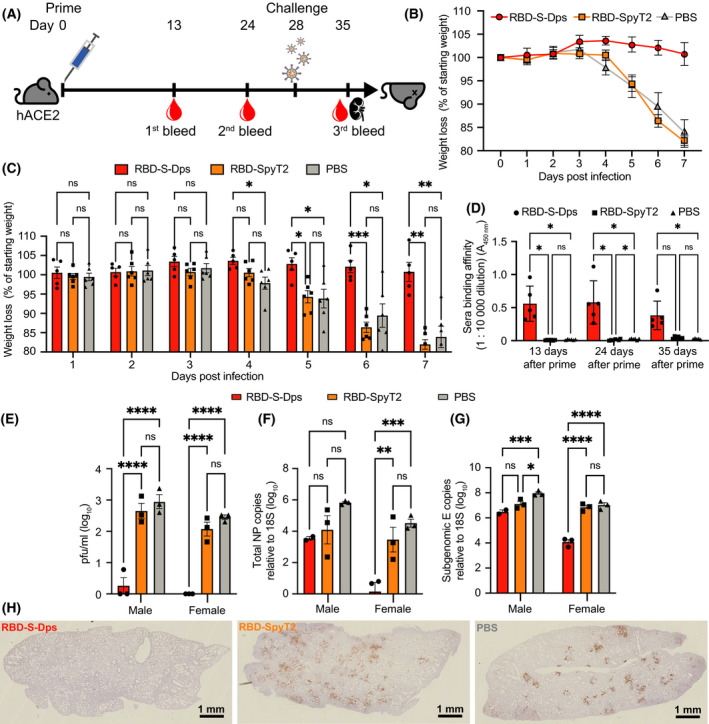
Single‐shot immunisation and Sars‐CoV‐2 challenge experiment using hACE2 mice. (A) Immunisation and challenge protocol. (B) K18‐hACE2 mice were immunised with 25 µg of RBD‐S‐Dps, RBD‐SpyT2 or given PBS, plus 10 µg CpG adjuvant. The animals were challenged on day 28 with 10^4^ PFU SARS‐CoV‐2 and changes in weight recorded. The animals in the PBS control group and those who had been given RBD‐SpyT2 showed the characteristic weight loss after four days post infection. RBD‐S‐Dps‐immunised mice showed no such weight loss. (C) Two‐way ANOVA tests on the weight changes between groups, as plotted in (B). Data are shown as the mean of three independent measurements with error bars representing the standard error of the mean. (D) Sera from days 13, 24 and 35 were tested for anti‐RBD antibodies by ELISA. Only RBD‐S‐Dps mice showed significant antibody. (E) Plaque assay using lung homogenates from mice culled 7 days postinfection. RBD‐S‐Dps‐immunised mice contained very low amounts of infectious SARS‐CoV‐2 in their lungs. (F, G) qPCR on RNA extracted from lung homogenates, using probes against *NP* or *E*, respectively. Both genomic and subgenomic RNA (gRNA, sgRNA) can be detected by *NP* qPCR, whilst *E* qPCR detects only subgenomic RNA. Two‐way ANOVA tests were carried out with significance levels of: *P* = < 0.05 (*), *P* = < 0.05 (**), *P* = < 0.005 (***), *P* = < 0.0005 (****). (H) Representative lung sections from animals (*n* = 6) taken seven days postchallenge, stained by immunohistology for SARS‐CoV‐2 NP protein.

Encouraged by these results, we wanted to know whether antigen display on our Dps scaffold would induce a sufficiently strong antibody response to protect animals from SARS‐CoV‐2 infection. We selected our most potent immunogen, RBD‐S‐Dps, and used it to immunise mice transgenic for human ACE2 (K18‐hACE2) [45]. As a single‐dose vaccination regime offers many downstream logistical and practical benefits, we opted to immunise only once and then challenge with SARS‐CoV‐2 on day 28 (Fig. 4A). We immunised subcutaneously six K18‐hACE2 mice with RBD‐S‐Dps, six with RBD‐SpyT2 and six with PBS (always three female and three male mice), each with 25 µg of the immunogens (except PBS control), plus CpG adjuvant. The anti‐Spike antibody response following immunisation was measured by ELISA on days 13 and 24 (before challenge) and on day 35 (7 days postchallenge). A strong anti‐Spike antibody titre was detected in RBD‐S‐Dps‐immunised mice, but almost none for either RBD‐SpyT2 or PBS (Fig. 4D). Antibody titres remained high for RBD‐S‐Dps at days 24 and 35.

On day 28, animals were challenged with 10^4^ PFU SARS‐CoV‐2. Mice in the PBS control and RBD‐SpyT2‐immunised groups began to show clinical signs of illness and a decline in body weight from day four postinfection (Fig. 4B), consistent with previous reports of infection in naïve animals [45]. In contrast, mice immunised with multimerised RBD‐S‐Dps maintained body weight until the day seven end point. There was a statistically significant difference in weights between the RBD‐S‐Dps‐immunised and PBS control groups from day four, and between RBD‐S‐Dps‐ and RBD‐SpyT2‐immunised mice from day five (Fig. 4C). There was no significant difference in weight loss between the RBD‐SpyT2‐immunised mice and PBS controls at any time point, suggesting that, unlike RBD‐S‐Dps, nonmultimerised RBD does not provide protection after only a single vaccination. All animals were culled on day seven postinfection and tissues collected for analysis. As mentioned, there were no significant changes in anti‐Spike antibody levels pre‐ versus postchallenge, indicating that mostly antibodies raised during the immunisation contributed to the immune response during the infection (Fig. 4D). SARS‐CoV‐2 infection of the lung was quantified by plaque assay and genomic and subgenomic qPCR, using probes against the viral genes *NP* and *E*, respectively. There were significantly lower levels of infectious virus in the lungs of mice immunised with RBD‐S‐Dps, compared to either RBD‐SpyT2 immunised or PBS control groups (Fig. 4E). A broadly similar pattern was observed when quantifying virus using probes against *NP* or *E* (Fig. 4F,G). However, we noted a marked difference in the amounts detected between male *vs* female mice. Female RBD‐S‐Dps‐immunised mice had significantly fewer genomic and subgenomic transcripts, compared to mice from other groups and their male equivalents (Fig. 4F,G and Fig. S3A,B). We attempted to correlate this with differences in antibody titres, but whilst there was a trend towards lower titres in male mice, particularly just before and just after the challenge, this was not significant (Fig. S3C). A larger group size would be needed to confirm this result. Despite these sex‐dependent differences in the qPCR data, the near‐absence of infectious virus in both male and female RBD‐S‐Dps immunised mice, as measured by plaque assay (Fig. S3D), suggests they were both highly protected. Finally, we examined the lungs of mice from the different groups for histopathological changes and for viral antigen expression using an anti‐NP antibody to reveal sites of replication (Fig. 4H, Fig. S4) and immune cell infiltration (Fig. S5). A detailed description is provided in the Appendix S1. In summary, lungs from RBD‐SpyT2‐immunised mice or PBS control mice showed substantial and widespread NP expression mainly in pneumocytes (Fig. 4H and Fig. S4), indicative of viral replication throughout the lobe and consistent with the high virus levels measured in these animals (Fig. 4E–G). There was also evidence of pneumocyte degeneration and syncytial cell formation, as has been reported in COVID‐19 cases postmortem [46]. Multifocal leukocyte infiltration was observed, particularly in PBS control animals, dominated by macrophages, followed by T cells (mainly CD4+ and lesser CD8+ cells), B cells and neutrophils (Figs S4 and S5). This is reminiscent of the hyperinflammation in postmortem reports of lethal COVID‐19 associated with immunopathology [47]. In contrast, the lungs of mice protected by multimerised RBD‐S‐Dps were either almost or entirely clear of NP expression (Fig. S4) and pathological changes (female mice), or showed only mild changes consistent with those observed in the PBS control animals (Fig. S5), and markedly reduced NP expression. Taken together, these data indicate that immunisation with RBD‐S‐Dps is highly protective against SARS‐CoV‐2 in hACE2‐expressing mice, even after a single dose, whilst monomeric RBD‐SpyT2 is not.

## Discussion

Here, we have shown that the ferritin‐like protein Dps from the hyperthermophilic archaeon *S. islandicus* possesses exceptional qualities as a SARS‐CoV‐2 subunit vaccine scaffold. We combined Dps with the SpyCatcher/SpyTag system in order to create a ‘plug‐and‐play’ system that allows the rapid and facile synthesis of highly stable multimeric subunit vaccines. Mixing the SpyCatcher‐Dps protein with any compatible SpyTag antigen leads to the assembly of highly monodisperse nanoparticles displaying exactly 12 antigens. Using this approach, we have produced subunit vaccines based on Spike, RBD or NP from SARS‐CoV‐2 and tested them in immunisation and viral challenge experiments. In each case, the Dps‐displayed antigens out‐performed their nonmultimerised equivalents and induced a more rapid and potent antibody response.

Subunit vaccines offer distinct advantages in cost, simplicity, production capacity, storage, transport and administration over nucleic‐acid based vaccines [48]. Principle amongst these considerations is stability, with currently used vaccines such as those from Pfizer‐BioNtech (Pfizer, New York City, New York, USA; BioNTech, Mainz, Germany), Moderna (Moderna, Cambridge, MA, USA), And Oxford‐AstraZeneca (Oxford University, Oxford, UK; AstraZeneca, Cambridge, UK) requiring a −80 °C or −20 °C cold‐chain. In countries without a highly developed logistical and medical infrastructure, this represents a significant impediment to vaccination. Whilst subunit vaccine development currently lags behind nucleic‐acid based equivalents, there is good evidence that such vaccines are nevertheless effective at inducing a protective response. SARS‐CoV‐2 RBD by itself [49] or in simple fusions such as to IgG Fc [50] has been shown to elicit SARS‐CoV‐2‐neutralising antibodies. Antigen multimerisation increases neutralising titres, for instance when using ferritin as a scaffold [23, 28, 29]. Our finding of a 3‐log increase in the neutralisation of live SARS‐CoV‐2 *in vitro* upon RBD multimerisation compares favourably with the 2‐log increase in neutralisation of Spike‐pseudotyped lentivirus observed by these earlier studies. However, neither study examined whether their multimerised scaffolds provided protection against SARS‐CoV‐2 in a live virus *in vivo* infection model. A live virus challenge experiment in mice was performed by Ma *et al*. [27], who tested their ferritin‐multimerised RBD vaccine using a prime/boost strategy and found that whilst monomeric RBD dramatically reduced viraemia upon SARS‐CoV‐2 challenge, multimerisation decreased it 1‐log further. A direct comparison with our data is difficult but the Dps scaffold presented here performs at least as good, if not better, as the difference in viraemia after vaccination with monomeric vs multimeric antigen we observed was in the order of several logs. Moreover, our data suggest that multimerisation is particularly beneficial when a single vaccine dose is used.

Nonferritin scaffolds have also been used to multimerise antigens. For instance, virus‐like icosahedral particles display 60 antigen copies (e.g. I3‐01) [51]. When fused directly to viral antigens [52], or using the SpyCatcher/SpyTag system [19, 53], the I3‐01 scaffold has been shown to induce a neutralising antibody response. Our scaffold differs from both ferritin and nonferritin scaffolds used previously to deliver SARS‐CoV‐2 immunogens in several important aspects. First, because we have used a thermostable protein it is intrinsically more stable, providing potential benefits to vaccine transport and storage and also to immunogen stability *in vivo*. Second, it is smaller than other scaffolds (< 10 nm vs > 10 nm for ferritin or 25 nm for the I3‐01 nanoparticle), making it an easier cargo for cellular uptake. Third, it displays fewer copies than ferritin or I3‐01 (12 vs 24 or 60, respectively), allowing the selection of higher‐affinity B cells and avoiding the activation of off‐target (and possibly cross‐reactive) B‐cell competitors [54]. Fourth, in contrast to *bona fide* ferritin scaffolds, the N and the C termini of Dps are both accessible on the outside of the sphere. This allows, at east in principle, for the conjugation of two discrete antigens onto a single scaffold, for example both SARS‐CoV‐2 Spike/RBD and NP to be displayed simultaneously.

Importantly, we have provided here data demonstrating the benefit of antigen multimerisation in inducing not just neutralising antibodies but an immune response capable of providing *in vivo* protection. In our SARS‐CoV‐2 challenge experiments, we found that RBD alone failed to protect mice, which displayed continued weight loss and high viral loads in the lungs. In contrast, our Dps‐based vaccine displaying RBD completely protected mice from SARS‐CoV‐2‐associated pneumonia and disease after only a single immunisation. We noted however a difference in Dps‐RBD induced protection between male and female mice, with the latter having lower viral loads and hardly any pulmonary changes. Trial data for both mRNA and vector‐based vaccines have not been disaggregated by sex but data on SARS‐CoV‐2 infection show that men are more at risk of severe adverse conditions, hospitalisation, and death [55, 56]. Our results support the consideration of sex as a variable in vaccine trials [57].

Further research is needed to develop the Dps scaffold into a *bona fide* vaccine for SARS‐CoV‐2 and other viruses. Replicating the robust neutralising antibody response and high level of protection achieved in mice from a single dose in primates and humans will be crucial, especially given that the hACE2 transgenic mouse model is not perfect. It does better recapitulate severe infection in humans than the Syrian hamster model but does not allow respiratory transmission [58]. The mouse model also has the advantage that it shows signs of neurological spread of the virus, something that is underappreciated in the human disease.

Whilst most studies of subunit vaccines have focused on antibodies, long‐lasting protection is likely to be dependent upon stimulating CD4+ and CD8+ T cell immunity [59, 60]. Data from current vaccine trials and roll‐outs have yet to be fully analysed but a correctly balanced T‐cell response appears associated with recovery from acute infection and the avoidance of hospitalisation and severe virus‐induced immunopathology [61]. Fortunately, the analysis of T‐cell epitopes from SARS‐CoV‐2 convalescents [62] provides a basis for engineering subunit vaccines specifically to engage both B and T cells. In this context, the ability of our Dps scaffold to display antigens at both termini may prove particularly beneficial. In addition to ensuring a well‐balanced immune response in humans, a more thorough investigation into the long‐term stability, storage, and reconstitution of lyophilised material is required to demonstrate that a Dps‐based vaccine is suitable for use in regions with limited infrastructure.

Future work notwithstanding, our data add to a body of evidence that subunit‐based vaccines represent a viable choice as a vaccine modality for SARS‐CoV‐2. Whilst other vaccine formats are significantly more advanced, subunit approaches such as Dps offer distinct advantages in simplicity of production, requiring no proprietary technology, robustness of material and potency of protection.

## Author contributions

Study design and conception: RS, ARA, JPS, LCJ, JL, with further study design by JAH, AO. Bacterial expression, purification, coupling reactions, EM analysis and stability assays: RS, with AG‐L (EM, lyophilisation) and LK (NP protein). Design of Spike and RBD constructs, protein production in HEK cells, purification: VTC. In vivo challenge experiment and downstream analysis: JJC and PS. Antibody analysis and neutralisation assays: MV and AA. Histopathology: AK. Manuscript preparation and editing: RS, JJC, PS, AK, JPS, LCJ, JL, with further editing by JAH, AO, and ARA.

## Supporting information


**Fig. S1.** A) Plasma stability assay. RBD‐S‐Dps was incubated with non‐heated human blood plasma for the amount of time indicated. SDS‐PAGE and Western blot against the histidine tag on the protein. Transferrin was used as loading control and also detected by Western blot. B) Quantification of the data in A) and a replicate experiment, with a one phase exponential decay fitted. The red line indicates the estimated half‐life of RBD‐S‐Dps of 39.7 h. C) SDS‐PAGE of RBD‐S‐Dps before and after lyophilisation. (Coomassie staining). D) The lyophilised sample from C) was diluted to two different concentrations to demonstrate monodispersity and subjected to negative stain electron microscopy (left 20 × dilution and right 100 × dilution). E) Antibody response in mice immunised with lyophilised RBD‐S‐DPS. Using the same immunisation protocol as in Fig 3 (see methods, only change: 2nd bleed at day 40), five mice in each group were injected with untreated, once‐ and twice‐lyophilised RBD‐S‐Dps. After collecting 1st and 2nd bleeds, ELISA assays on pooled samples per experimental group detected and quantified antibodies against Spike‐SpyT2 (assayed in duplicate). Error bars represent the standard error of the mean (n = 2).
**Fig. S2.** A, B): Vero cells expressing ACE2 and TMPRSS2 were infected with SARS‐CoV‐2 in the presence of serial dilutions of antisera. Viral replication was then determined after 24 h by RT‐qPCR using probes for gRNA (A) or sgRNA (B). Each point represents sera from an individual mouse. C) ELISpot measuring IFNg production in splenocytes from mice 74 days post‐immunisation upon stimulation with a peptide library covering Spike protein. Error bars depict the mean +/‐ standard error of the mean.
**Fig. S3.** Mice were immunised with RBD‐S‐Dps, RBD‐SpyT2 or given PBS control on day 1 and then challenged with SARS‐CoV‐2 on day 28.
**Fig. S4.** Lung, left lobe, K‐18 hACE2 mice at day 7 post infection. Histological changes and SARS‐CoV‐2 antigen expression.
**Fig. S5.** Lung, K18‐hACE2 mice. Composition of the inflammatory infiltrates.
**Table S1.** Amino acid sequences of the proteins used in this work.
**Appendix S1.** Supplemental results.

## Data Availability

The data that support the findings of this study are available from the corresponding authors (lcj@mrc-lmb.cam.ac.uk; jyl@mrc-lmb.cam.ac.uk) upon reasonable request.

## References

[1] Cucinotta D and Vanelli M (2020) WHO declares COVID‐19 a pandemic. Acta Biomed 91, 157–160. 32191675 10.23750/abm.v91i1.9397 PMC7569573

[2] The New York Times, Coronavirus Vaccine Tracker (The New York Times) (2021).

[3] World Health Organization, COVID‐19 situation reports (2021).

[4] Pal M , Berhanu G , Desalegn C and Kandi V (2020) Severe acute respiratory syndrome coronavirus‐2 (SARS‐CoV‐2): an update. Cureus 12, e7423. 32337143 10.7759/cureus.7423 PMC7182166

[5] Zeng W , Liu G , Ma H , Zhao D , Yang Y , Liu M , Mohammed A , Zhao C , Yang Y , Xie J *et al*. (2020) Biochemical characterization of SARS‐CoV‐2 nucleocapsid protein. Biochem Biophys Res Comm 527, 618–623. 32416961 10.1016/j.bbrc.2020.04.136 PMC7190499

[6] Ke Z , Oton J , Qu K , Cortese M , Zila V , McKeane L , Nakane T , Zivanov J , Neufeldt CJ , Cerikan B *et al*. (2020) Structures and distributions of SARS‐CoV‐2 spike proteins on intact virions. Nature 588, 498–502. 32805734 10.1038/s41586-020-2665-2 PMC7116492

[7] Shang J , Wan Y , Luo C , Ye G , Geng Q , Auerbach A and Li F (2020) Cell entry mechanisms of SARS‐CoV‐2. Proc Natl Acad Sci USA 117, 11727–11734. 32376634 10.1073/pnas.2003138117 PMC7260975

[8] Huang Y , Yang C , Xu XF , Xu W and Liu SW (2020) Structural and functional properties of SARS‐CoV‐2 spike protein: potential antivirus drug development for COVID‐19. Acta Pharmacol Sin 41, 1141–1149. 32747721 10.1038/s41401-020-0485-4 PMC7396720

[9] Papa G , Mallery DL , Albecka A , Welch LG , Cattin‐Ortola J , Luptak J , Paul D , McMahon HT , Goodfellow IG , Carter A *et al*. (2021) Furin cleavage of SARS‐CoV‐2 Spike promotes but is not essential for infection and cell‐cell fusion. PLoS Pathog 17, e1009246. 33493182 10.1371/journal.ppat.1009246 PMC7861537

[10] Seydoux E , Homad LJ , MacCamy AJ , Parks KR , Hurlburt NK , Jennewein MF , Akins NR , Stuart AB , Wan YH , Feng J *et al*. (2020) Characterization of neutralizing antibodies from a SARS‐CoV‐2 infected individual. BioRxiv [PREPRINT]. 10.1016/j.immuni.2020.06.001 PMC7276322 32561270

[11] Mahase E (2020) Covid‐19: what do we know about the late stage vaccine candidates? BMJ 371, m4576. 33234507 10.1136/bmj.m4576

[12] Thompson MG , Burgess JL , Naleway AL , Tyner HL , Yoon SK , Meece J , Olsho LEW , Caban‐Martinez AJ , Fowlkes A , Lutrick K *et al*. (2021) Interim estimates of vaccine effectiveness of BNT162b2 and mRNA‐1273 COVID‐19 vaccines in preventing SARS‐CoV‐2 infection among health care personnel, first responders, and other essential and frontline workers – eight U.S. locations, December 2020‐March 2021. MMWR Morb Mortal Wkly Rep 70, 495–500. 33793460 10.15585/mmwr.mm7013e3 PMC8022879

[13] Mahase E (2021) Covid‐19: Novavax vaccine efficacy is 86% against UK variant and 60% against South African variant. BMJ 372, n296. 33526412 10.1136/bmj.n296

[14] Davies NG , Abbott S , Barnard RC , Jarvis CI , Kucharski AJ , Munday JD , Pearson CAB , Russell TW , Tully DC , Washburne AD *et al*. (2021) Estimated transmissibility and impact of SARS‐CoV‐2 lineage B.1.1.7 in England. Science 372, eabg3055. 33658326 10.1126/science.abg3055 PMC8128288

[15] Ferreira I , Datir R , Papa G , Kemp S , Meng B , Rakshit P , Singh S , Pandey R , Ponnusamy K , Radhakrishnan VS *et al*. (2021) SARS‐CoV‐2 B.1.617 emergence and sensitivity to vaccine‐elicited antibodies. BioRxiv 2021.05.08.443253 [PREPRINT].

[16] Kupferschmidt K (2021) New mutations raise specter of “immune escape”. Science 371, 329–330. 33479129 10.1126/science.371.6527.329

[17] Zhang W , Davis BD , Chen SS , Sincuir Martinez JM , Plummer JT and Vail E (2021) Emergence of a novel SARS‐CoV‐2 variant in Southern California. JAMA 325, 1324–1326. 33571356 10.1001/jama.2021.1612 PMC7879386

[18] Bottermann M , Foss S , van Tienen LM , Vaysburd M , Cruickshank J , O'Connell K , Clark J , Mayes K , Higginson K , Hirst JC *et al*. (2018) TRIM21 mediates antibody inhibition of adenovirus‐based gene delivery and vaccination. Proc Natl Acad Sci USA 115, 10440–10445. 30209217 10.1073/pnas.1806314115 PMC6187179

[19] Tan TK , Rijal P , Rahikainen R , Keeble AH , Schimanski L , Hussain S , Harvey R , Hayes JWP , Edwards JC , McLean RK *et al*. (2021) A COVID‐19 vaccine candidate using SpyCatcher multimerization of the SARS‐CoV‐2 spike protein receptor‐binding domain induces potent neutralising antibody responses. Nat Commun 12, 542. 33483491 10.1038/s41467-020-20654-7 PMC7822889

[20] Wang W , Huang B , Zhu Y , Tan W and Zhu M (2021) Ferritin nanoparticle‐based SARS‐CoV‐2 RBD vaccine induces a persistent antibody response and long‐term memory in mice. Cell Mol Immunol 18, 749–751. 33580169 10.1038/s41423-021-00643-6 PMC7880661

[21] Dalvie NC , Rodriguez‐Aponte SA , Hartwell BL , Tostanoski LH , Biedermann AM , Crowell LE , Kaur K , Kumru O , Carter L , Yu J *et al*. (2021) Engineered SARS‐CoV‐2 receptor binding domain improves immunogenicity in mice and elicits protective immunity in hamsters. BioRxiv 2021.03.03.433558 [PREPRINT]. 10.1073/pnas.2106845118 PMC8463846 34493582

[22] Gu M , Torres JL , Greenhouse J , Wallace S , Chiang C‐I , Jackson AM , Porto M , Kar S , Li Y , Ward AB *et al*. (2021) One dose of COVID‐19 nanoparticle vaccine REVC‐128 provides protection against SARS‐CoV‐2 challenge at two weeks post immunization. BioRxiv 2021.04.02.438218 [PREPRINT]. 10.1080/22221751.2021.1994354 PMC8567933 34651563

[23] He L , Lin X , Wang Y , Abraham C , Sou C , Ngo T , Zhang Y , Wilson IA and Zhu J (2021) Single‐component, self‐assembling, protein nanoparticles presenting the receptor binding domain and stabilized spike as SARS‐CoV‐2 vaccine candidates. Science. Advances 7, eabf1591. 10.1126/sciadv.abf1591 PMC7978432 33741598

[24] Joyce MG , King HAD , Naouar IE , Ahmed A , Peachman KK , Cincotta CM , Subra C , Chen RE , Thomas PV , Chen WH *et al*. (2021) Efficacy of a broadly neutralizing SARS‐CoV‐2 ferritin nanoparticle vaccine in nonhuman primates. BioRxiv [PREPRINT].

[25] Kalathiya U , Padariya M , Fahraeus R , Chakraborti S and Hupp TR (2021) Multivalent display of SARS‐CoV‐2 spike (RBD domain) of COVID‐19 to nanomaterial, protein ferritin nanocages. Biomolecules 11, 297. 33671255 10.3390/biom11020297 PMC7923090

[26] Koenig PA , Das H , Liu H , Kummerer BM , Gohr FN , Jenster LM , Schiffelers LDJ , Tesfamariam YM , Uchima M , Wuerth JD *et al*. (2021) Structure‐guided multivalent nanobodies block SARS‐CoV‐2 infection and suppress mutational escape. Science 371, eabe6230. 33436526 10.1126/science.abe6230 PMC7932109

[27] Ma X , Zou F , Yu F , Li R , Yuan Y , Zhang Y , Zhang X , Deng J , Chen T , Song Z *et al*. (2020) Nanoparticle vaccines based on the receptor binding domain (RBD) and heptad repeat (HR) of SARS‐CoV‐2 elicit robust protective immune responses. Immunity 53, 1315–1330 e9. 33275896 10.1016/j.immuni.2020.11.015 PMC7687490

[28] Powell AE , Zhang K , Sanyal M , Tang S , Weidenbacher PA , Li S , Pham TD , Pak JE , Chiu W and Kim PS (2021) A single immunization with spike‐functionalized ferritin vaccines elicits neutralizing antibody responses against SARS‐CoV‐2 in mice. ACS Cent Sci 7, 183–199. 33527087 10.1021/acscentsci.0c01405 PMC7805605

[29] Saunders KO , Lee E , Parks R , Martinez DR , Li D , Chen H , Edwards RJ , Gobeil S , Barr M , Mansouri K *et al*. (2021) Neutralizing antibody vaccine for pandemic and pre‐emergent coronaviruses. Nature 594, 553–559. 33971664 10.1038/s41586-021-03594-0 PMC8528238

[30] Xiang Y , Nambulli S , Xiao Z , Liu H , Sang Z , Duprex WP , Schneidman‐Duhovny D , Zhang C and Shi Y (2020) Versatile and multivalent nanobodies efficiently neutralize SARS‐CoV‐2. Science 370, 1479–1484. 33154108 10.1126/science.abe4747 PMC7857400

[31] Gauss GH , Benas P , Wiedenheft B , Young M , Douglas T and Lawrence CM (2006) Structure of the DPS‐like protein from *Sulfolobus solfataricus* reveals a bacterioferritin‐like dimetal binding site within a DPS‐like dodecameric assembly. Biochemistry 45, 10815–10827. 16953567 10.1021/bi060782u PMC1815386

[32] Pickering S , Betancor G , Galao RP , Merrick B , Signell AW , Wilson HD , Kia Ik MT , Seow J , Graham C , Acors S *et al*. (2020) Comparative assessment of multiple COVID‐19 serological technologies supports continued evaluation of point‐of‐care lateral flow assays in hospital and community healthcare settings. PLoS Pathog 16, e1008817. 32970782 10.1371/journal.ppat.1008817 PMC7514033

[33] Wrapp D , Wang N , Corbett KS , Goldsmith JA , Hsieh CL , Abiona O , Graham BS and McLellan JS (2020) Cryo‐EM structure of the 2019‐nCoV spike in the prefusion conformation. Science 367, 1260–1263. 32075877 10.1126/science.abb2507 PMC7164637

[34] Chang VT , Spooner RA , Crispin M and Davis SJ (2015) Glycan remodeling with processing inhibitors and lectin‐resistant eukaryotic cells. Methods Mol Biol 1321, 307–322. 26082231 10.1007/978-1-4939-2760-9_21

[35] Chang VT , Fernandes RA , Ganzinger KA , Lee SF , Siebold C , McColl J , Jonsson P , Palayret M , Harlos K , Coles CH *et al*. (2016) Initiation of T cell signaling by CD45 segregation at “close contacts”. Nat Immunol 17, 574–582. 26998761 10.1038/ni.3392 PMC4839504

[36] Elegheert J , Behiels E , Bishop B , Scott S , Woolley RE , Griffiths SC , Byrne EFX , Chang VT , Stuart DI , Jones EY *et al*. (2018) Lentiviral transduction of mammalian cells for fast, scalable and high‐level production of soluble and membrane proteins. Nat Protoc 13, 2991–3017. 30455477 10.1038/s41596-018-0075-9 PMC6364805

[37] Aricescu AR , Lu W and Jones EY (2006) A time‐ and cost‐efficient system for high‐level protein production in mammalian cells. Acta Crystallogr D Biol Crystallogr 62, 1243–1250. 17001101 10.1107/S0907444906029799

[38] Morecroft JA and Thomas MH (1988) Radionuclide ejection fraction. Br J Surg 75, 188. 10.1002/bjs.1800750239 3349318

[39] Patterson EI , Prince T , Anderson ER , Casas‐Sanchez A , Smith SL , Cansado‐Utrilla C , Solomon T , Griffiths MJ , Acosta‐Serrano A , Turtle L *et al*. (2020) Methods of inactivation of SARS‐CoV‐2 for downstream biological assays. J Infect Dis 222, 1462–1467. 32798217 10.1093/infdis/jiaa507 PMC7529010

[40] Davidson AD , Williamson MK , Lewis S , Shoemark D , Carroll MW , Heesom KJ , Zambon M , Ellis J , Lewis PA , Hiscox JA *et al*. (2020) Characterisation of the transcriptome and proteome of SARS‐CoV‐2 reveals a cell passage induced in‐frame deletion of the furin‐like cleavage site from the spike glycoprotein. Genome Med 12, 68. 32723359 10.1186/s13073-020-00763-0 PMC7386171

[41] Wölfel R , Corman VM , Guggemos W , Seilmaier M , Zange S , Müller MA , Niemeyer D , Jones TC , Vollmar P , Rothe C *et al*. (2020) Virological assessment of hospitalized patients with COVID‐2019. Nature 581, 465–469. 32235945 10.1038/s41586-020-2196-x

[42] Brune KD and Howarth M (2018) New routes and opportunities for modular construction of particulate vaccines: stick, click, and glue. Front Immunol 9, 1432. 29997617 10.3389/fimmu.2018.01432 PMC6028521

[43] Zakeri B , Fierer JO , Celik E , Chittock EC , Schwarz‐Linek U , Moy VT and Howarth M (2012) Peptide tag forming a rapid covalent bond to a protein, through engineering a bacterial adhesin. Proc Natl Acad Sci USA 109, E690–E697. 22366317 10.1073/pnas.1115485109 PMC3311370

[44] Bruun TUJ , Andersson A‐MC , Draper SJ and Howarth M (2018) Engineering a rugged nanoscaffold to enhance plug‐and‐display vaccination. ACS Nano 12, 8855–8866. 30028591 10.1021/acsnano.8b02805 PMC6158681

[45] Zheng J , Wong LR , Li K , Verma AK , Ortiz ME , Wohlford‐Lenane C , Leidinger MR , Knudson CM , Meyerholz DK , McCray PB Jr *et al*. (2021) COVID‐19 treatments and pathogenesis including anosmia in K18‐hACE2 mice. Nature 589, 603–607. 33166988 10.1038/s41586-020-2943-z PMC7855185

[46] Bussani R , Schneider E , Zentilin L , Collesi C , Ali H , Braga L , Volpe MC , Colliva A , Zanconati F , Berlot G *et al*. (2020) Persistence of viral RNA, pneumocyte syncytia and thrombosis are hallmarks of advanced COVID‐19 pathology. EBioMedicine 61, 103104. 33158808 10.1016/j.ebiom.2020.103104 PMC7677597

[47] Schurink B , Roos E , Radonic T , Barbe E , Bouman CSC , de Boer HH , de Bree GJ , Bulle EB , Aronica EM , Florquin S *et al*. (2020) Viral presence and immunopathology in patients with lethal COVID‐19: a prospective autopsy cohort study. Lancet Microbe 1, e290–e299. 33015653 10.1016/S2666-5247(20)30144-0 PMC7518879

[48] Pollet J , Chen WH and Strych U (2021) Recombinant protein vaccines, a proven approach against coronavirus pandemics. Adv Drug Deliv Rev 170, 71–82. 33421475 10.1016/j.addr.2021.01.001 PMC7788321

[49] Yang J , Wang W , Chen Z , Lu S , Yang F , Bi Z , Bao L , Mo F , Li X , Huang Y *et al*. (2020) A vaccine targeting the RBD of the S protein of SARS‐CoV‐2 induces protective immunity. Nature 586, 572–577. 32726802 10.1038/s41586-020-2599-8

[50] Liu Z , Xu W , Xia S , Gu C , Wang X , Wang Q , Zhou J , Wu Y , Cai X , Qu D *et al*. (2020) RBD‐Fc‐based COVID‐19 vaccine candidate induces highly potent SARS‐CoV‐2 neutralizing antibody response. Signal Transduct Target Ther 5, 282. 33247109 10.1038/s41392-020-00402-5 PMC7691975

[51] Hsia Y , Bale JB , Gonen S , Shi D , Sheffler W , Fong KK , Nattermann U , Xu C , Huang PS , Ravichandran R *et al*. (2016) Design of a hyperstable 60‐subunit protein dodecahedron [corrected]. Nature 535, 136–139. 27309817 10.1038/nature18010 PMC4945409

[52] Walls AC , Fiala B , Schafer A , Wrenn S , Pham MN , Murphy M , Tse LV , Shehata L , O'Connor MA , Chen C *et al*. (2020) Elicitation of potent neutralizing antibody responses by designed protein nanoparticle vaccines for SARS‐CoV‐2. Cell 183, 1367–1382 e17. 33160446 10.1016/j.cell.2020.10.043 PMC7604136

[53] Cohen AA , Gnanapragasam PNP , Lee YE , Hoffman PR , Ou S , Kakutani LM , Keeffe JR , Wu HJ , Howarth M , West AP *et al*. (2021) Mosaic nanoparticles elicit cross‐reactive immune responses to zoonotic coronaviruses in mice. Science 371, 735–741. 33436524 10.1126/science.abf6840 PMC7928838

[54] Kato Y , Abbott RK , Freeman BL , Haupt S , Groschel B , Silva M , Menis S , Irvine DJ , Schief WR and Crotty S (2020) Multifaceted effects of antigen valency on B cell response composition and differentiation in vivo. Immunity 53, 548–563, e8. 32857950 10.1016/j.immuni.2020.08.001 PMC7451196

[55] Klein SL , Dhakal S , Ursin RL , Deshpande S , Sandberg K and Mauvais‐Jarvis F (2020) Biological sex impacts COVID‐19 outcomes. PLoS Pathog 16, e1008570. 32569293 10.1371/journal.ppat.1008570 PMC7307725

[56] Scully EP , Haverfield J , Ursin RL , Tannenbaum C and Klein SL (2020) Considering how biological sex impacts immune responses and COVID‐19 outcomes. Nat Rev Immunol 20, 442–447. 32528136 10.1038/s41577-020-0348-8 PMC7288618

[57] Bischof E , Wolfe J and Klein SL (2020) Clinical trials for COVID‐19 should include sex as a variable. J Clin Investig 130, 3350–3352. 32392184 10.1172/JCI139306 PMC7324163

[58] Jia W , Wang J , Sun B , Zhou J , Shi Y and Zhou Z (2021) The mechanisms and animal models of SARS‐CoV‐2 infection. Front Cell Dev Biol 9, 578825. 33987176 10.3389/fcell.2021.578825 PMC8111004

[59] Sauer K and Harris T (2020) An effective COVID‐19 vaccine needs to engage T cells. Front Immunol 11, 581807. 33117391 10.3389/fimmu.2020.581807 PMC7549399

[60] Zuo J , Dowell AC , Pearce H , Verma K , Long HM , Begum J , Aiano F , Amin‐Chowdhury Z , Hallis B , Stapley L *et al*. (2021) Robust SARS‐CoV‐2‐specific T cell immunity is maintained at 6 months following primary infection. Nat Immunol 22, 620–626. 33674800 10.1038/s41590-021-00902-8 PMC7610739

[61] Chen Z and John Wherry E (2020) T cell responses in patients with COVID‐19. Nat Rev Immunol 20, 529–536. 32728222 10.1038/s41577-020-0402-6 PMC7389156

[62] Nelde A , Bilich T , Heitmann JS , Maringer Y , Salih HR , Roerden M , Lubke M , Bauer J , Rieth J , Wacker M *et al*. (2021) SARS‐CoV‐2‐derived peptides define heterologous and COVID‐19‐induced T cell recognition. Nat Immunol 22, 74–85. 32999467 10.1038/s41590-020-00808-x

