# Supplementary material for: Single‐dose immunisation with a multimerised SARS‐CoV‐2 receptor binding domain (RBD) induces an enhanced and protective response in mice
Source: FEBS Lett. 2021 Aug 22;595(18):2323–40. doi: 10.1002/1873-3468.14171 (PMC8426897; doi:10.1002/1873-3468.14171)
Supplement: Supplementary file 1 — Fig. S1. A) Plasma stability assay. RBD‐S‐Dps was incubated with non‐heated human blood plasma for the amount of time indicated. SDS‐PAGE and Western blot against the histidine tag on the protein. Transferrin was used as loading control and also detected by Western blot. B) Quantification of the data in A) and a replicate experiment, with a one phase exponential decay fitted. The red line indicates the estimated half‐life of RBD‐S‐Dps of 39.7 h. C) SDS‐PAGE of RBD‐S‐Dps before and after lyophilisation. (Coomassie staining). D) The lyophilised sample from C) was diluted to two different concentrations to demonstrate monodispersity and subjected to negative stain electron microscopy (left 20 × dilution and right 100 × dilution). E) Antibody response in mice immunised with lyophilised RBD‐S‐DPS. Using the same immunisation protocol as in Fig 3 (see methods, only change: 2nd bleed at day 40), five mice in each group were injected with untreated, once‐ and twice‐lyophilised RBD‐S‐Dps. After collecting 1st and 2nd bleeds, ELISA assays on pooled samples per experimental group detected and quantified antibodies against Spike‐SpyT2 (assayed in duplicate). Error bars represent the standard error of the mean (n = 2). Fig. S2. A, B): Vero cells expressing ACE2 and TMPRSS2 were infected with SARS‐CoV‐2 in the presence of serial dilutions of antisera. Viral replication was then determined after 24 h by RT‐qPCR using probes for gRNA (A) or sgRNA (B). Each point represents sera from an individual mouse. C) ELISpot measuring IFNg production in splenocytes from mice 74 days post‐immunisation upon stimulation with a peptide library covering Spike protein. Error bars depict the mean +/‐ standard error of the mean. Fig. S3. Mice were immunised with RBD‐S‐Dps, RBD‐SpyT2 or given PBS control on day 1 and then challenged with SARS‐CoV‐2 on day 28. Fig. S4. Lung, left lobe, K‐18 hACE2 mice at day 7 post infection. Histological changes and SARS‐CoV‐2 antigen expression. Fig. S5. Lung, K18‐hA [file FEB2-595-2323-s001.pdf]

SUPPORTING INFORMATION

Single-dose immunisation with a multimerised SARS-CoV-2 receptor binding domain (RBD) induces an enhanced and protective response in mice

Ralf Salzer, Jordan J. Clark, Marina Vaysburd, Veronica T. Chang, Anna Albecka, Leo Kiss, Parul Sharma, Andres Gonzalez Llamazares, Anja Kipar, Julian A. Hiscox Andrew Owen, A. Radu Aricescu, James P. Stewart, Leo C. James, and Jan Löwe

SUPPLEMENTAL RESULTS

Detailed description of the lung histology in RBS-D-Dps-immunised mice subsequently challenged with SARS-Cov-2 (Fig. 4A):

Mice immunised with PBS control. All animals showed a mild to moderate increase in interstitial cellularity and multifocal extensive areas of consolidation due to macrophage and lymphocyte infiltration, with a few neutrophils and with abundant activated type II cells, occasional syncytial cells and some degenerate cells and moderate mesothelial cell activation above the affected parenchymal area (Suppl. Fig. 4A). The changes were associated with extensive viral antigen expression in type I and type II pneumocytes both in consolidated areas and in alveoli without inflammatory changes. Occasional macrophages in the infiltrate also appeared to express viral antigen (Suppl. Fig. 4B). Macrophages (Iba1+) were the dominant cells in the infiltrates (Suppl. Fig. 5A), followed by numerous T cells with a relatively high proportion and CD4 positive cells and less CD8 positive cells (Suppl. Fig. 5C, E, G), and a moderate number of B cells (Suppl. Fig. 5I). There were also areas where alveoli exhibited type II cell activation and desquamation, with desquamation of alveolar macrophages and some neutrophils in the lumen. In addition, mild to moderate mononuclear vasculitis (mainly arteritis) was seen (Suppl. Fig. 4A). Also, the infiltrate was dominated by macrophages, followed by T cells (CD4 positive cells and less CD8 positive cells) and fewer B cells.

Mice immunised with monomeric RBD-SpyT2. All animals showed a mild to moderate increase in interstitial cellularity and multifocal extensive areas of consolidation similar in composition and extent to those seen in the PBS-control mice (Suppl. Fig. 4C). Viral antigen was detected in multiple variably-sized foci, in type I and II pneumocytes and in macrophages within and close to consolidated areas (Suppl. Fig. 4D).

Mice immunised with multimerised RBD-S-Dps. The three female animals showed minimal histological changes in the lungs (Suppl. Fig. 4E). Besides a very mild increase in interstitial cellularity, with rare T cells (both CD4 and CD8 positive cells) and B cells, one animal showed scattered small focal leukocyte aggregates. Viral antigen expression was restricted to a few macrophages in the leukocyte aggregates in the latter animal, while a second had positive pneumocyte in an alveolus; in the third lung, viral antigen was not detected (Suppl. Fig. 4F). In male animals, multifocal inflammatory infiltrates with viral antigen expression and mild

vasculitis similar to the other two groups, but substantially less extensive were seen (Suppl. Fig. 4G, H). Also here, macrophages were the dominant cells in the focal infiltrates (Suppl. Fig. 5B), followed by the T cells (Suppl. Fig. 5D). The T cell population showed a mild shift in composition, as now CD8-positive cells were as numerous or more frequent than CD4-positive cells (Suppl. Fig. 5F-H). CD8-positive cells were also seen within alveolar lumina (Suppl. Fig. 5B inset). B cells were found in moderate numbers in the infiltrates, in one animal they also formed peribronchiolar aggregates (Suppl. Fig. 5J).

SUPPLEMENTAL FIGURES & TABLES

Supplemental Figure 1

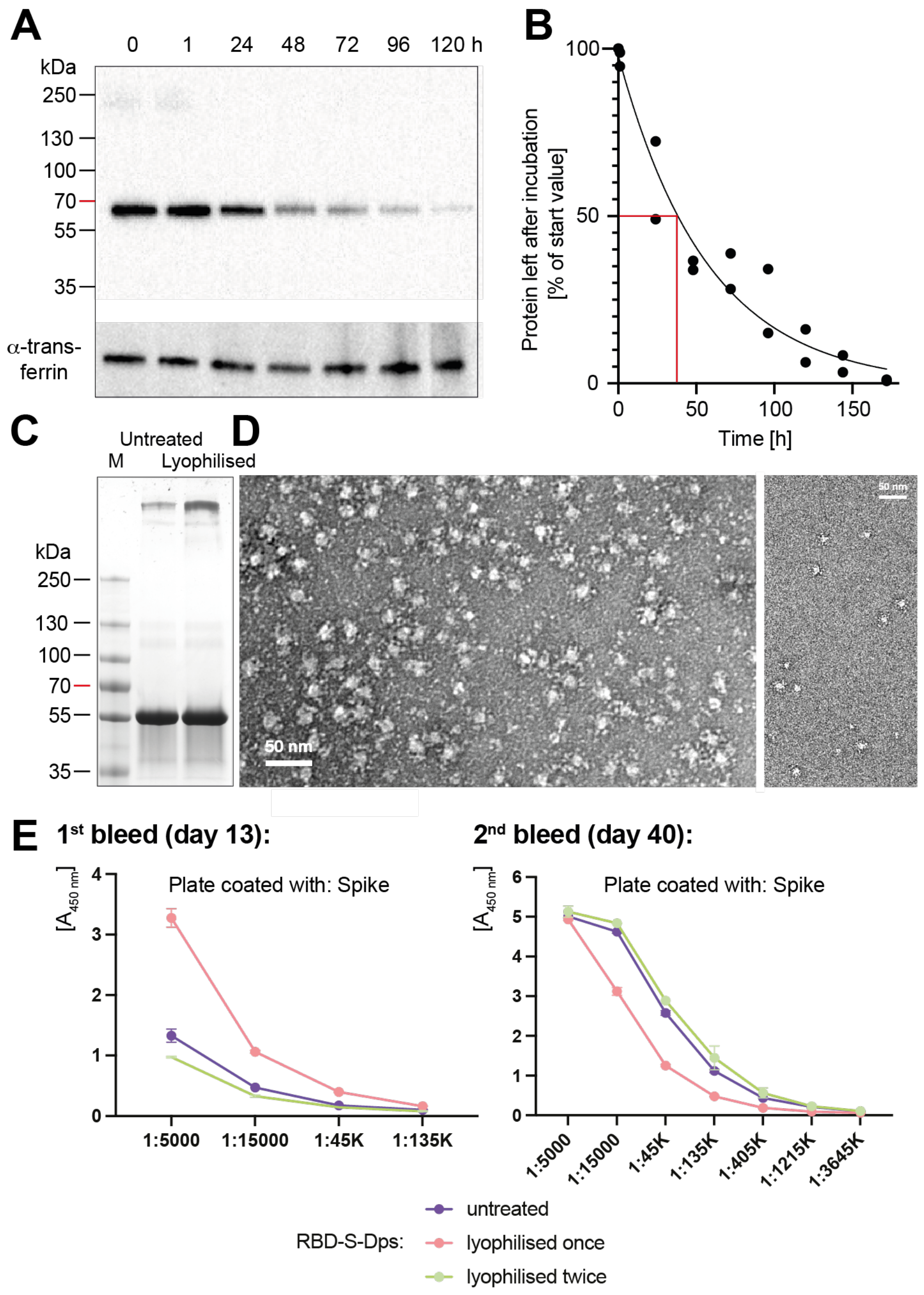

A) Plasma stability assay. RBD-S-Dps was incubated with non-heated human blood plasma for the amount of time indicated. SDS-PAGE and Western blot against the histidine tag on the protein. Transferrin was used as loading control and also detected by Western blot. **B)** Quantification of the data in A) and a replicate experiment, with a one phase exponential decay

fitted. The red line indicates the estimated half-life of RBD-S-Dps of 39.7 h. **C)** SDS-PAGE of RBD-S-Dps before and after lyophilisation. (Coomassie staining). **D)** The lyophilised sample from C) was diluted to two different concentrations to demonstrate monodispersity and subjected to negative stain electron microscopy (left 20 x dilution and right 100 x dilution). **E)** Antibody response in mice immunised with lyophilised RBD-S-DPS. Using the same immunisation protocol as in Fig 3 (see methods, only change: 2nd bleed at day 40), five mice in each group were injected with untreated, once- and twice-lyophilised RBD-S-Dps. After collecting 1st and 2nd bleeds, ELISA assays on pooled samples per experimental group detected and quantified antibodies against Spike-SpyT2 (assayed in duplicate). Error bars represent the standard error of the mean (n = 2).

Supplemental Figure 2

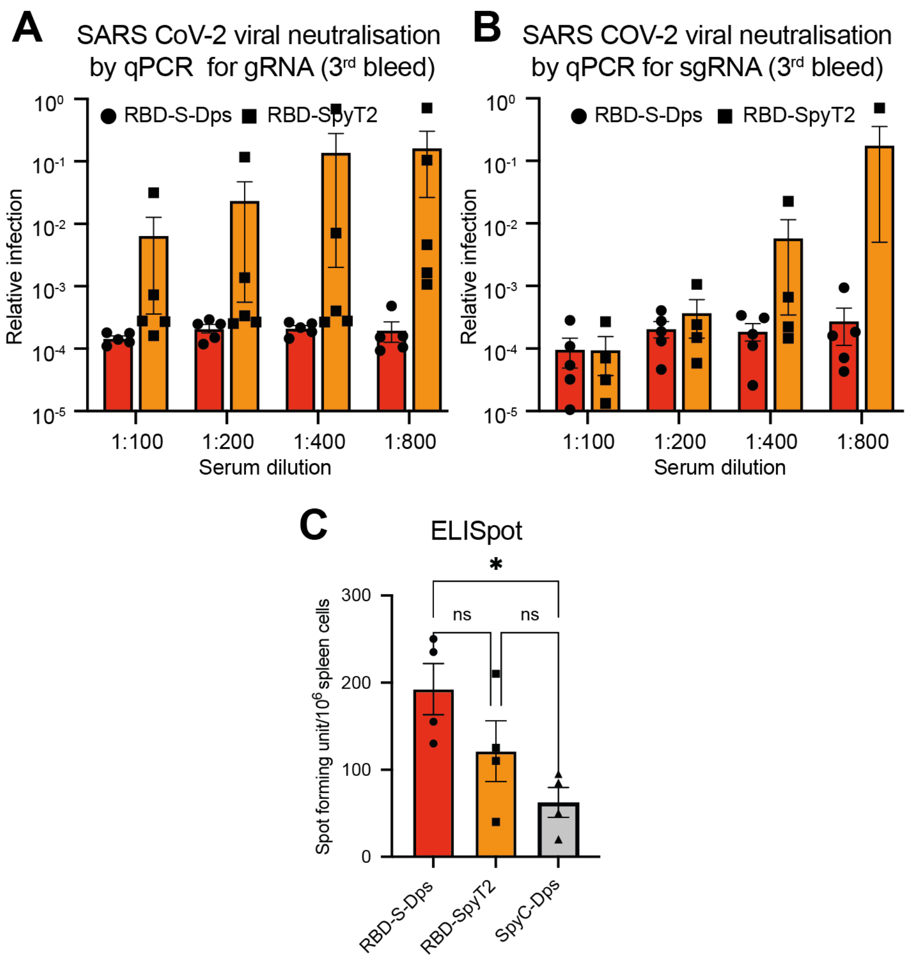

A, B): Vero cells expressing ACE2 and TMPRSS2 were infected with SARS-CoV-2 in the presence of serial dilutions of antisera. Viral replication was then determined after 24 h by RT-qPCR using probes for gRNA **(A)** or sgRNA **(B)**. Each point represents sera from an individual mouse. **C)** ELISpot measuring IFNγ production in splenocytes from mice 74 days post-immunisation upon stimulation with a peptide library covering Spike protein. Error bars depict the mean +/- standard error of the mean.

Supplemental Figure 3

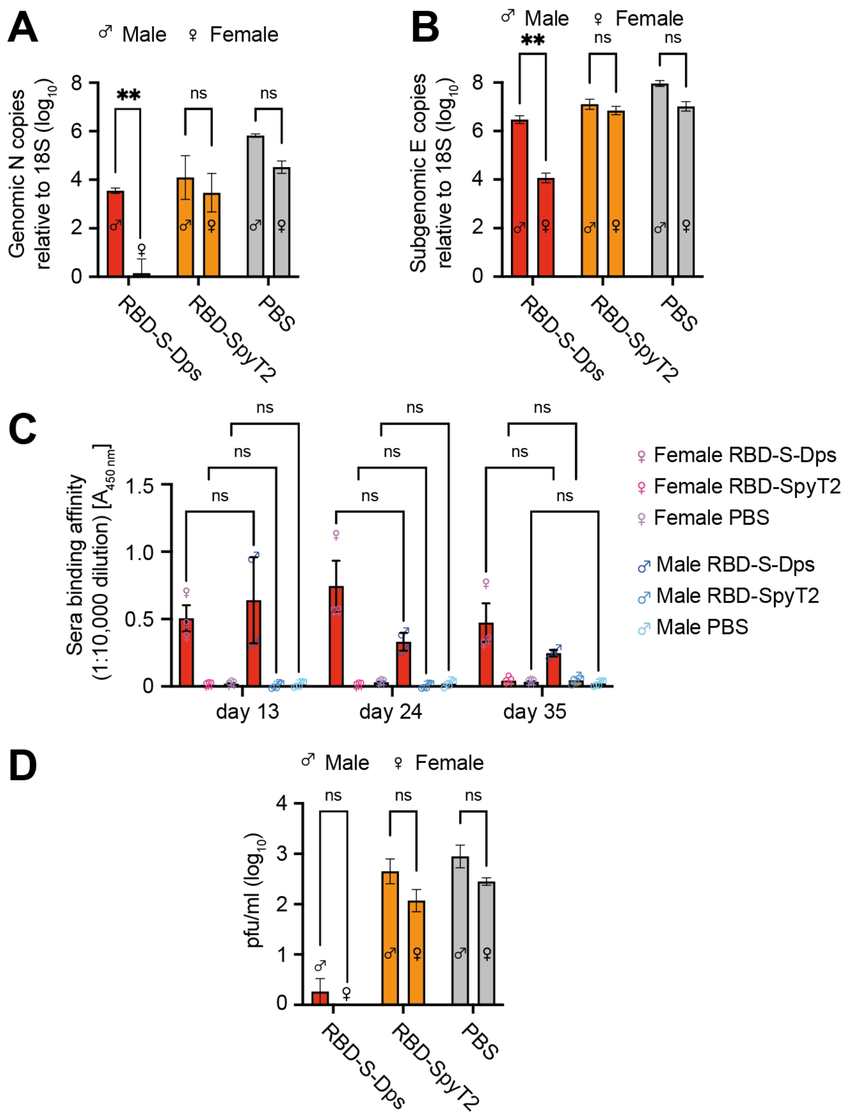

Mice were immunised with RBD-S-Dps, RBD-SpyT2 or given PBS control on day 1 and then challenged with SARS-CoV-2 on day 28. **A & B)** Genomic and subgenomic (gRNA, sgRNA) qPCR on RNA extracted from lung homogenates, using probes against *NP* or *E*, respectively. **C)** Sera from days 13, 24 and 35 were tested for anti-RBD antibodies by ELISA. Two-way ANOVA tests show that there are non-significant differences between male and female antibody responses. **D)** Plaque assay using lung homogenates from mice culled seven days post-infection.

Supplemental Figure 4

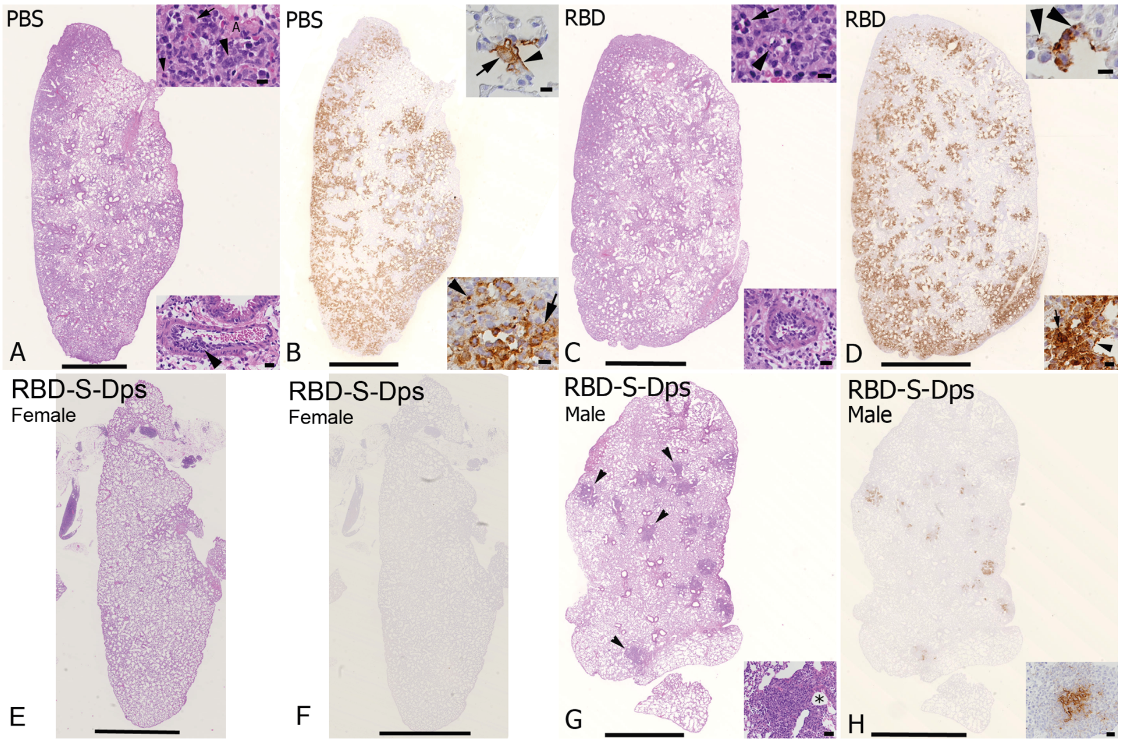

Lung, left lobe, K-18 hACE2 mice at day 7 post infection. Histological changes and SARS-CoV-2 antigen expression. **A, B: mice injected with PBS control. A)** Overview of the lung lobe, with multifocal extensive cell rich consolidated areas. Bar = 2.5 mm. Inset top: Consolidated area with activated type II pneumocyte (small arrow), syncytial cells (large arrowhead) and infiltrating neutrophil (small arrowhead). A – alveolus. Bar = 10 µm. Inset bottom: Artery with leukocyte infiltration of the wall (arrowhead; arteritis). Bar = 20 µm. HE stain. **B.** Extensive SARS-CoV-2 antigen expression is seen in multifocal patchy areas within and close to consolidated areas, in pneumocytes and occasional macrophages. Bar = 2.5 mm. Inset top: Alveolus with viral antigen expression in type I (arrowhead) and type II (arrow) pneumocyte. Inset bottom: Consolidated area with viral antigen expression in macrophages (arrow) and degenerate cells (arrowhead). Immunohistology, hematoxylin counterstain. Bars = 10 µm. **C, D: Monomeric RBD-SpyT2-immunised mice. C)** Overview of the lung lobe, with multifocal extensive cell rich consolidated areas. Bar = 2.5 mm. Inset top: Consolidated area with several neutrophils (arrowhead) and occasional necrotic cells (arrowhead). Bar = 10 µm. Inset bottom: Artery with leukocyte infiltration of the wall (arrowhead; arteritis) and mild periarterial edema. Bar = 20 µm. HE stain. **D.** Extensive SARS-CoV-2 antigen expression is seen in multifocal patchy

areas within and close to consolidated areas, in pneumocytes and occasional macrophages. Bar = 2.5 mm. Inset top: Alveolus with viral antigen expression in pneumocytes of which some are degenerate (arrowheads). Inset bottom: Consolidated area with viral antigen expression in macrophages (arrow) and type I pneumocyte (arrowhead). Immunohistology, hematoxylin counterstain. Bars = 10 µm. **E-H: Multimerised RBD-S-Dps immunised mice**. **E, F.** Female animal. **E.** Overview of the lung lobe. The histological changes are restricted to focal areas of mildly increased interstitial cellularity. HE stain. Bar = 2.5 mm. **F**. There is no evidence of viral antigen expression. Immunohistology, hematoxylin counterstain. **G, H.** Male animal. Bar = 2.5 mm. **G.** Overview of the lung lobe, with several small, randomly distributed peribronchiolar cell rich areas (arrowheads). Bar = 2.5 mm. Inset: Closer view of focal cell rich area (asterisk: bronchiole). Bar = 50 µm. HE stain. **H**. SARS-CoV-2 antigen expression is restricted to the cell rich focal areas, in pneumocytes and occasional macrophages (inset). Immunohistology, hematoxylin counterstain. Bar = 20 µm.

Supplemental Figure 5

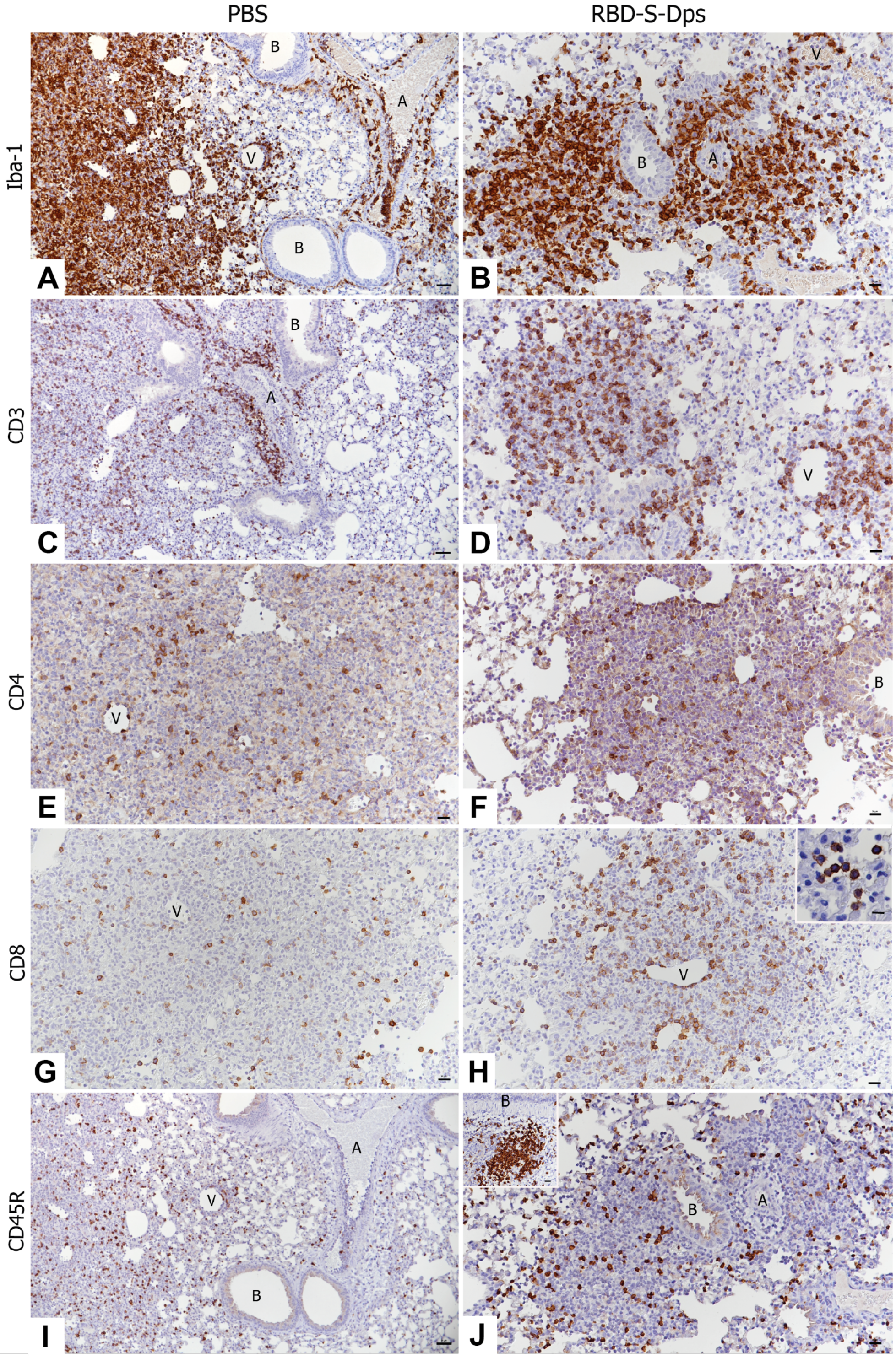

Lung, K18-hACE2 mice. Composition of the inflammatory infiltrates. **A, B:** staining for macrophages (Iba1+). **A)** PBS-control animal. Macrophages are the dominant infiltrating cells

in the consolidated areas and in the vasculitis. A – artery with infiltration of the wall. V – vein with infiltration of the wall. B – bronchiole. Bar = 50 µm. **B)** RBD-S-Dps animal, male. Macrophages are the dominant infiltrating cells in the focal infiltrates. A – artery. V – vein. B – bronchiole. Bar = 20 µm. **C, D:** staining for T cells (CD3+). **C)** PBS-control animal. T cells are numerous in the consolidated areas and in the vasculitis. A – artery with infiltration of the wall and perivascular T cell accumulation. B – bronchiole. Bar = 50 µm. **D)** RBD-S-Dps animal, male. T cells are numerous in the focal infiltrates. V – vein. Bar = 20 µm. **E, F:** staining for CD4. **E)** PBS-control animal. Within the infiltrates, CD4 positive cells are numerous. V – vein. Bar = 20 µm. **F)** RBD-S-Dps animal, male. Within the infiltrates, CD4 positive cells are present in moderate number. B – bronchiole. Bar = 20 µm. **G, H:** staining for CD8. **G)** PBS-control animal. CD8 positive cells are less numerous. V – vein. Bar = 20 µm. **H)** RBD-S-Dps animal, male. CD8 positive cells are more abundant. V – vein. Bar = 20 µm. Inset: CD8 positive cells are also present in the lumen of several alveoli. Bar = 10 µm. **I, J:** staining for B cells (CD45R/B220+). **I)** PBS-control animal. B cells are observed in moderate numbers in the consolidated areas and are rare in the vasculitis. A – artery with infiltration of the wall. V – vein with infiltration of the wall. B – bronchiole. Bar = 50 µm. **J)** RBD-S-Dps animal, male. B cells (CD45R/B220+) are observed in moderate numbers in the focal infiltrates. A – artery. B – bronchiole. Bar = 20 µm. Inset: Focal peribronchial (B) B cell aggregate. Bar = 20 µm. Immunohistology, haematoxylin counterstain.

Supplemental Table 1

Amino acid sequences of the proteins used in this work. Signal sequences for secretion in mammalian cells are indicated in red.

Protein	Amino acid sequence
SpyC-Dps	MGHHHHHHGGSDSATHIKFSKRDEDGKELAGATMELRDSSGKTISTWISDGQVKDFYL<br>YPGKYTFVETAAPDGYEVATAITFTVNEQGQVTVNGKATKGDAHIGSEGSSGGQDKPK<br>EEPKVVGVEVLEKSGLDVKKLIEKLVKATAAEFTTYYYYTILRMHLTGMEGEGLKEIA<br>EDARLEDRLHFELMTQRIYELGGNLPRDIRQLADLSACADAYLPENWKDPKEILKVLL<br>EAEQCAIRTWKEVCDMTYGKDPRTYDLAQRILQEEIEHEAWFLELLYGRPSGHFRRSY<br>PGEGPFSRKSRYE
SpyT2-NP (Nucleocapsid Protein)	MAHHHHHHGGSVPTIVMVDAYKRYKGGSGGSGGNTASWFTALTQHGKEDLKFPRGQGV<br>PINTNSSPDDQIGYYRRATRRIRGGDGKMKDLSPRWYFYYLGTGPEAGLPYGANKDGI<br>IWVATEGALNTPKDHIGTRNPANNAAIVLQLPQGTTLPKGFYAEGSRGGSQASSRSSS<br>RSRNSSRNSTPGSSRGTSPARMAGNGGDAALALLLLDRLNQLESKMSGKGQQQQGQTV<br>TKKSAAEASKKPRQKRTATKAYNVTQAFGRRGPEQTQGNFGDQELIRQGTDYKHWPQI<br>AQFAPSASAFFGMSRIGMEVTPSGTWLTYTGAIKLDDKDPNFKDQVILLNKHIDAYKT<br>FP
RBD-SpyT2	(MGILPSPGMPALLSLVSLLSVLLMGCVA)<br>ETGITNLCPFGEVFNATRFASVYAWNRKRISNCVADYSVLYNSASFSTFKCYGVSPTK<br>LNDLCFTNVYADSFVIRGDEVRQIAPGQTGKIADYNYKLPDDFTGCVIAWNSNNLDSK<br>VGGNYNYLYRLFRKSNLKPFERDISTEIYQAGSTPCNGVEGFNCYFPLQSYGFQPTNG<br>VGYQPYRVVVLSFELLHAPATVCGPKKGTGGSGGSGLNDIFEAQKIEWHEGGSHHHHH<br>HHHGGSGGSGGSVPTIVMVDAYKRYK
Spike	(MGILPSPGMPALLSLVSLLSVLLMGCVA)<br>ETGVNLTTRTQLPPAYTNSFTRGVYYPDKVFRSSVLHSTQDLFLPFFSNVTWFHAIHV<br>SGTNGTKRFDNPVLPFNDGVYFASTEKSNIIRGWIFGTTLDSKTQSLLIVNNATNVVI<br>KVCEFQFCNDPFLGVYYHKNNKSWMESEFRVYSSANNCTFEYVSQPFLMDLEGKQGNF<br>KNLREFVFKNIDGYFKIYSKHTPINLVRDLPQGFSALEPLVDLPIGINITRFQTLLAL<br>HRSYLTPGDSSSGWTAGAAAYYVGYLQPRTFLLKYNENGTITDAVDCALDPLSETKCT<br>LKSFTVEKGIYQTSNFRVQPTESIVRFPNITNLCPFGEVFNATRFASVYAWNRKRISN<br>CVADYSVLYNSASFSTFKCYGVSPTKLNDLCFTNVYADSFVIRGDEVRQIAPGQTGKI<br>ADYNYKLPDDFTGCVIAWNSNNLDSKVGGNYNYLYRLFRKSNLKPFERDISTEIYQAG<br>STPCNGVEGFNCYFPLQSYGFQPTNGVGYQPYRVVVLSFELLHAPATVCGPKKSTNLV<br>KNKCVNFNFNGLTGTGVLTESNKKFLPFQQFGRDIADTTDAVRDPQTLEILDITPCSF<br>GGVSVITPGTNTSNQVAVLYQDVNCTEVPVAIHADQLTPTWRVYSTGSNVFQTRAGCL<br>IGAEHVNNSYECDIPIGAGICASYQTQTNSPGSASSVASQSIIAYTMSLGAENSVAYS<br>NNSIAIPTNFTISVTTEILPVSMTKTSVDCTMYICGDSTECSNLLLQYGSFCTQLNRA<br>LTGIAVEQDKNTQEVFAQVKQIYKTPPIKDFGGFNFSQILPDPSKPSKRSFIEDLLFN<br>KVTLADAGFIKQYGDCLGDIAARDLICAQKFNGLTVLPPLLTDEMIAQYTSALLAGTI<br>TSGWTFGAGAALQIPFAMQMAYRFNGIGVTQNVLYENQKLIANQFNSAIGKIQDSLSS<br>TASALGKLQDVVNQNAQALNTLVKQLSSNFGAISSVLNDILSRLDPPEAEVQIDRLIT<br>GRLQSLQTYVTQQLIRAAEIRASANLAATKMSECVLGQSKRVDFCGKGYHLMSFPQSA<br>PHGVVFLHVTYVPAQEKNFTTAPAICHDGKAHFPREGVFVSNGTHWFVTQRNFYEPQI<br>ITTDNTFVSGNCDVVIGIVNNTVYDPLQPELDSFKEELDKYFKNHTSPDVDLGDISGI<br>NASVVNIQKEIDRLNEVAKNLNESLIDLQELGKYEQGSGYIPEAPRDGQAYVRKDGEW<br>VLLSTFLGRSGGSLEVLFQGPGGSGLNDIFEAQKIEWHEGGSHHHHHH
Spike-SpyT2	(MGILPSPGMPALLSLVSLLSVLLMGCVA)<br>ETGVNLTTRTQLPPAYTNSFTRGVYYPDKVFRSSVLHSTQDLFLPFFSNVTWFHAIHV<br>SGTNGTKRFDNPVLPFNDGVYFASTEKSNIIRGWIFGTTLDSKTQSLLIVNNATNVVI<br>KVCEFQFCNDPFLGVYYHKNNKSWMESEFRVYSSANNCTFEYVSQPFLMDLEGKQGNF

	KNLREFVFKNIDGYFKIYSKHTPINLVRDLPQGFSALEPLVDLPIGINITRFQTLLAL<br>HRSYLTPGDSSSGWTAGAAAYYVGYLQPRTFLLKYNENGTITDAVDCALDPLSETKCT<br>LKSFTVEKGIYQTSNFRVQPTESIVRFPNITNLCPFGEVFNATRFASVYAWNRKRISN<br>CVADYSVLYNSASFSTFKCYGVSPTKLNDLCFTNVYADSFVIRGDEVRQIAPGQTGKI<br>ADYNYKLPDDFTGCVIAWNSNNLDSKVGGNYNYLYRLFRKSNLKPFERDISTEIYQAG<br>STPCNGVEGFNCYFPLQSYGFQPTNGVGYQPYRVVVLSFELLHAPATVCGPKKSTNLV<br>KNKCVNFNFNGLTGTGVLTESNKKFLPFQQFGRDIADTTDAVRDPQTLEILDITPCSF<br>GGVSVITPGTNTSNQVAVLYQDVNCTEVPVAIHADQLTPTWRVYSTGSNVFQTRAGCL<br>IGAEHVNNSYECDIPIGAGICASYQTQTNSPGSASSVASQSIIAYTMSLGAENSVAYS<br>NNSIAIPTNFTISVTTEILPVSMTKTSVDCTMYICGDSTECSNLLLQYGSFCTQLNRA<br>LTGIAVEQDKNTQEVFAQVKQIYKTPPIKDFGGFNFSQILPDPSKPSKRSFIEDLLFN<br>KVTLADAGFIKQYGDCLGDIAARDLICAQKFNGLTVLPPLLTDEMIAQYTSALLAGTI<br>TSGWTFGAGAALQIPFAMQMAYRFNGIGVTQNVLYENQKLIANQFNSAIGKIQDSLSS<br>TASALGKLQDVVNQNAQALNTLVKQLSSNFGAISSVLNDILSRLDPPEAEVQIDRLIT<br>GRLQSLQTYVTQQLIRAAEIRASANLAATKMSECVLGQSKRVDFCGKGYHLMSFPQSA<br>PHGVVFLHVTYVPAQEKNFTTAPAICHDGKAHFPREGVFVSNGTHWFVTQRNFYEPQI<br>ITTDNTFVSGNCDVVIGIVNNTVYDPLQPELDSFKEELDKYFKNHTSPDVDLGDISGI<br>NASVVNIQKEIDRLNEVAKNLNESLIDLQELGKYEQGSGYIPEAPRDGQAYVRKDGEW<br>VLLSTFLGRSGTGGSGGSGLNDIFEAQKIEWHEGGSHHHHHHHHGGSGGSGGSVPTIV<br>MVDAYKRYK
